# Computer-Assisted Diagnosis of the Sleep Apnea-Hypopnea Syndrome: A Review

**DOI:** 10.1155/2015/237878

**Published:** 2015-07-21

**Authors:** Diego Alvarez-Estevez, Vicente Moret-Bonillo

**Affiliations:** ^1^Sleep Center, Medisch Centrum Haaglanden, 2512 VA The Hague, Netherlands; ^2^Laboratory for Research and Development in Artificial Intelligence (LIDIA), Department of Computer Science, University of A Coruña, 15071 A Coruña, Spain

## Abstract

Automatic diagnosis of the Sleep Apnea-Hypopnea Syndrome (SAHS) has become an important area of research due to the growing interest in the field of sleep medicine and the costs associated with its manual diagnosis. The increment and heterogeneity of the different techniques, however, make it somewhat difficult to adequately follow the recent developments. A literature review within the area of computer-assisted diagnosis of SAHS has been performed comprising the last 15 years of research in the field. Screening approaches, methods for the detection and classification of respiratory events, comprehensive diagnostic systems, and an outline of current commercial approaches are reviewed. An overview of the different methods is presented together with validation analysis and critical discussion of the current state of the art.

## 1. Clinical Background

Sleep Apnea-Hypopnea Syndrome (SAHS) is one of the most common disorders affecting sleep, characterized by the repeated occurrence of involuntary episodes of total or partial reduction in patient's respiration during the night [1]. Several studies have been carried around the world during the last years, which estimate that the prevalence of SAHS is between the 3% and the 7% of the adult population [2, 3].

Patients suffering from SAHS present involuntary respiratory pauses that repeats throughout the night. The duration of these nocturnal respiratory events (from now on apneic events) is variable and it depends on the concrete patient; however to be clinically significant, the duration must be of at least 10 seconds, normally not exceeding the 2 minutes. Common duration of the apneic event, though, is usually about 20 to 40 seconds. One main distinction can be made within the apneic event attending to the associated reduction in the airflow. For that purpose the baseline breathing can be determined which is defined as a period of regular breathing with stable oxygen levels [4]. Thus, in a broad sense a hypopnea is defined as a respiratory pause meeting the duration criteria with an associated reduction around 30–50% with respect to the baseline breathing. The exact definition however highly depends on the concrete reference [5]. In the case of an apnea the associated reduction is more pronounced reaching about 90% or even total breathing cessation. Last standard definitions by the American Academy of Sleep Medicine (AASM) can be consulted in [6].

Respiratory pauses associated with the presence of an apneic event are also usually accompanied by a drop in the oxygen saturation levels. This fall is proportional to the causing airflow reduction. As a consequence, the lack of oxygen in arterial blood usually triggers an autonomic response increasing the alertness level of the individual which often causes neurophysiological awakening [7, 8]. These associated microarousals break up the normal sleep structure preventing a refreshing rest. Therefore daytime sleepiness is usual in apneic patients, impacting on their social, working, and family life. In addition, one of the main problems of this disease is that patients are usually unaware of their own symptoms. That contributes also to the fact that most of the SAHS patients are currently underdiagnosed [9].

In 1983 Guilleminault et al. [10] described cardiac arrhythmias and behavior disorders related to SAHS. This description was followed by several reports searching for cardiac arrhythmia, hypertension, cerebrovascular accidents, and sudden death as sequels of SAHS. Nowadays SAHS is associated with an increasing risk of suffering cardiac and cerebral infarct, high arterial pressure, arrhythmias, and, in general, several dysfunctions of the cardiorespiratory system [11–14].

The standard diagnostic procedure to determine the presence of SAHS requires a polysomnographic test to be done during the night. This test is normally carried out in the sleep units of the medical centers, and it involves the recording of several physiological signals during the night, both respiratory and neurophysiological. The resulting recording, namely, polysomnographic recording or PSG, is then visually analyzed offline by the medical specialists. From this analysis, one important derived measure is the Apnea-Hypopnea Index (AHI), which is calculated as the number of apneic events (either apnea or hypopnea) present in the PSG per hour of sleep and is used as objective indicator to quantify the SAHS severity in a subject. This index is also used as fundamental measure involved in the diagnosis of the syndrome in which cut-off values ranging between 5 and 15 are usually established as thresholds above which the AHI is regarded as clinically relevant in the diagnostic procedure [9]. Assessment of the AHI implies manual revision of the PSG recording, considering all evidences present in the respiratory signals and interpreting them according to contextual information of remaining PSG signals.

Given the complexity of the scoring process, mainly because of the length of a full-night PSG recording, as well as the amount and the diversity of signals involved, the analysis of the PSG represents a big cost both in time and in effort for the clinician. This high cost associated with the manual visual review of the PSG can eventually degenerate into quality loss of the analysis due to the accumulated tiredness throughout the revision and because of the complexity of the task itself. From the point of view of hospital's administration, it may also represent a waste of the personnel resources who could be dedicated to other affairs. In general the consequence is a saturation of the current sleep units, not being able to support the demand for the analysis. Eventually, all the previous causes an elevation in the economic costs associated with the diagnosis of SAHS.

In this regard, the development of systems that automate, at least in part, the analysis of the PSG represents great saving in terms of time, money, and effort, facilitating the clinician's task in a great extent and optimizing his/her time. Indeed, in the ideal scenario of a system being able to completely—and correctly—perform the automatic analysis of the PSG, the use of supporting tools for the diagnosis would help the scorer to focus his/her attention only on the real relevant information (e.g., the AHI). Indeed, ultimately the physician's task could be reduced to the sole task of checking and/or confirming the results of the computer analysis.

## 2. Motivation

In a revision made in 2000 by Penzel and Conradt about computer systems for the recording and the analysis of the sleep, there was a reference to the four tasks that a system of such characteristics should fulfill [15].

The first task is that systems should replace conventional paper chart recorders. The objective is to save paper and minimize archiving space requirements without losing the raw data. The second task is documentation. With the computer-based system, a technician should be able to enter and store all necessary notes and observations made during the nocturnal recording. The third task is supporting the evaluation of sleep and cardiorespiratory functions. In this regard, an automatic sleep scoring system should use its computational power to support the PSG scoring task. Specifically, according to [15] the system should be able to analyze electroencephalography (EEG), electrooculography (EOG), and electromyography (EMG) in terms of sleep stages; respiration, snoring, and oxygen saturation (SaO_2_) for the diagnosis of sleep related breathing disorders; and EMG tibialis in terms of limb movement disorders. Other parameters recorded in a sleep laboratory, such as body temperature, electrocardiography (ECG), blood pressure, capnography, and esophageal pressure (Pes), may require additional analysis. On the other hand, the system should support visual evaluation as an alternative to automatic analysis, and it should allow editing of the results of the automatic scoring. The fourth task is reporting. The computer-based system should help in the generation of a final report containing the relevant information for the diagnosis, and it should include an advanced filing system to archive the report as well as the data in a structured way. This will allow the sleep laboratory to keep track of patients and to recall reports when needed for revision [15].

Nowadays almost nobody doubts that tasks one, two, and four are quite covered already, since the incorporation of the Health Management Information Systems into the hospitals, including the sleep labs, is already a reality. It is regarding the task number three, the one relative to the capabilities of computer programs to perform the automatic analysis of the PSG, however, where these systems still present shortages and, as a consequence, where most of the research effort is concentrated at present.

The emerging importance of the sleep medicine during the last years has also contributed to an increasing demand in the diagnosis of SAHS and its associated treatment. As a consequence, the number of PSG studies has also increased, which, in the lack of a simpler and more precise test, still today continues to be the only standard procedure for the diagnosis of SAHS. Portable monitoring has emerged in the last years, aiming at mitigating the problematic effects of the limited resources at the hospital centers. It has been mentioned already, however, that visual analysis of the PSG is a costly, tedious, and time-consuming task with an impact on the working life of the medical specialists, who have to devote great part of their time to the scoring of the night registrations. With this precedent, the interest grows toward the development of automatic systems to aid the physician in the diagnosis of SAHS.

These two factors (increasing demands in the analysis and the still present shortages of the current analysis systems) have favored an increment of the research activity in the field in the last years. As a consequence, research in the development of computer methods for the analysis of sleep, and specifically for the diagnosis of SAHS, is today an important open area interest. The increasing number of research publications (and the lack of comprehensive reviews in this respect) demands an integrative effort to be done. This has motivated the authors of this paper to perform a literature review, trying to group together the most recent research efforts in this respect and to make them available as a single resource to serve the scientific community.

This review is thus intended to perform an update of the current state of the art in the field of computer-aided diagnosis of SAHS. The aim especially is to cover recent approaches and advances in the field during approximately the last 15 years. Ultimately it is the intention of the authors with this review to provide the interested reader with a useful resource in order to keep track of the most recent advances in the field.

## 3. Methods

This paper is aimed at covering approaches in the field of SAHS which are to some extent supported by automatic computer-based processes helping the diagnosis. This includes as well mathematical models and/or algorithm descriptions that can be eventually implemented into a computer program. Bibliography not assessing the evaluation of the presence of SAHS in the subject will not be considered (will be excluded from the evaluation). This includes a considerable number of works related to computer approaches to support sleep analysis but not specifically related to SAHS (e.g., automatic sleep scoring) which are not considered here. On the other hand, to keep the review attainable, the focus will be constrained to methods involving analysis of the respiratory function in accordance with the current standard clinical procedures. In this respect, the last version of the AASM manual for scoring of sleep and associated events is considered as in [6]. That means approaches must operate using, at least, a subset of the PSG signals which are proposed in the AASM manual for such purposes (see section VIII, Respiratory Rules in [6]). This rules out approaches relying, for example, on the use of questionnaires, clinical prediction models, and/or analysis of nonstandard biomedical signals for the diagnosis of SAHS such as the ECG or the EEG. Studies in which the only computer support refers to the recording and/or the visualization of the digital signals are not considered under this review. Therefore studies referring to devices in which diagnosis is exclusively based on visual human scoring are also omitted from the analysis. Finally, although some references may be included, it is not the objective of this paper to cover SAHS treatment, including devices or methods dealing with Continuous Positive Airway Pressure (CPAP).

Given the previous context, analysis of the relevant bibliography has been derived in part from our own experience and research in the field, plus literature search which has been carried out using different well-known search engines including ScienceDirect, IEEE explorer, PubMed, and Google Scholar. Search terms included combinations of the words “apn(o)eic,” “apn(o)ea,” “intelligent,” “OSAS,” “OSAHS,” “OSA,” “SAHS,” “monitoring,” “diagnosis,” “automatic,” “computer,” and “analysis.” That returned a first group of works which was subsequently redefined by careful revision of the corresponding abstracts in order to filter out studies nonrelevant for the purposes of this review. The resulting group of selected works was, in addition, supplementary expanded by a thorough search within the reference lists of the eligible studies, and the relevant studies were selected and included in the analysis.

With regard to the time coverage, the authors placed special emphasis on covering the most recent published literature over the last 15 years (from January 1999 up to December 2013) although some references to preceding works may be present when considered relevant or for introductory purposes. Only peer-reviewed literature in English was included in the study. In general, for all the methods analyzed within this review, discussion is based exclusively on the data published in the corresponding works. Also, when analyzing literature regarding commercial devices, data available from manufacturers on their websites, in product brochures, or published in non-peer-reviewed journals is not considered as well.

To structure the discussion, approximations analyzed under this review are organized into several categories as described below. For each category the related works are introduced following a descriptive approach and, at the end of each section, a summary table is presented for quick reference that contains an overview of the different approaches analyzed in the corresponding category. On the other hand, in an attempt to provide quantitative data for validation and comparison purposes, a second summary table within each section is provided. Structure of these tables may slightly vary from section to section according to the topic of the discussion and therefore specific description of their related structure is provided in the corresponding section. Nonetheless it is common for all the validation tables that the widely used measures of sensitivity and specificity (when available in the referenced study) are used to characterize the results. In this respect, it is worth saying that if several results are reported in the referenced study (e.g., due to different configurations of the computer method), the best performance will be always selected as the general criterion. The best performance is here interpreted as the point that maximizes the following value:* (sensitivity + specificity)/2*. This is equivalent to the area under the corresponding ROC curve with one-operation point. It is therefore an optimistic criterion for which it corresponds to the best operation point.

Organization of the review is structured as follows. In subsequent Section 4, general analysis of the current state of the art in the field of automatic diagnosis of SAHS is performed. In order to structure the different approximations, the discussion is divided into different subsections.  Section 4.1 comprises screening approaches performing over the biomedical signals recorded in the PSG.  Section 4.2 deals with approximations in which detection of the apneic event is performed individually throughout the recording. This section is further divided according to the reference used for the detection. Specifically approximations are structured comprising processing of the pulse oximetry signal (Section 4.2.1), alternative single-channel respiratory signals (Section 4.2.2), and multichannel analysis of the respiration.  Section 4.3 is dedicated to approximations specialized in the classification of the apneic event according to its origin (i.e., central, obstructive, or mixed). In the last part of Section 4 (Section 4.4), the analysis is focused on comprehensive approaches covering respiration as well as neurophysiological information.

The analysis is expanded in Section 5 to briefly discuss validation results from publications regarding commercial approximations. General discussion is performed in Section 6 which will carry out a critical analysis of the different approximations described throughout this review. Finally, concluding remarks are assessed in Section 7.

## 4. State of the Art in the Computer-Based Diagnosis of SAHS

Throughout this section the objective of the discussion is to reflect the current research state on the different methods and techniques developed for the computer assisted diagnosis of SAHS. As stated above, in order to structure the discussion, the different approximations have been divided into different subsections and four well-differenced types of approximations can be distinguished, namely, approaches comprising SAHS screening approaches, methods for the individual detection of the apneic events, methods focused on the classification of the apneic event type, and comprehensive approaches.

### 4.1. Screening Approaches for SAHS Estimation

Due to the complexity and the elevated costs associated with the PSG test, much of the current research interest in the diagnosis of SAHS is focused on reducing the necessity of (at first) submitting the patient to conduct night-attended in-hospital monitoring. For that purpose, the resulting approximations are normally aimed at substituting PSG outcomes, for example, AHI, by means of the calculation of a simpler supplementary measure which can be interpreted as a preliminary evaluation of SAHS severity and is meant to fulfill a screening function. In this respect, computer methods targeting a screening task comprise, as a minimum, the classification of the subject either as normal or as apneic. Some methods, in addition, may also attempt to predict (through regression) the associated severity index. Many approaches can be found in the literature with such characteristics, but, as stated in the Introduction, we here focus on methods that, to some extent, involve the recording and/or the subsequent analysis of subset of the respiratory signals contained in the PSG.

An overview of the different screening approaches analyzed throughout this section is shown in Table 1. Subsequently, Table 2 summarizes validation data of the corresponding approaches. For interpretation of the contents of Table 2, description of the different columns is done as follows.

Column 1 in Table 2 indicates the bibliographic reference. Columns 2 to 5 include study population details, respectively, the database identification, the number and gender of the participants, the age of the population, and the reference severity index. Columns 6 and 7 describe the gold standard to establish the clinical SAHS severity reference. The performance of the computer screening method is evaluated using this gold standard.

In this respect, column 6 describes the reference test, which can either be a retrospective clinical diagnosis or an overnight recording test, in which case, the type of test performed (AASM level) as well as whether the test was performed in the same night or in a different night (as with respect to the recording analyzed by the computer method) is indicated. Column 7, on the other hand, specifies the event definition followed by the gold standard. Indeed when AHI is used as the quantitative severity reference (most of the cases), it is reasonable to think that such index would be influenced by the specific scoring rules and event definitions followed in each study. Specifically, while from the beginning there has been a reasonable agreement on the definition of apnea, this has not been the case for the scoring of hypopneas [5, 41]. Column 7 therefore contains (when present in the study) a reference to the definition followed for the scoring of these events. Due to space restrictions, though, for full details on the event definition (e.g., for apnea scoring or information on the sensors), the reader is still committed to checking the full text within the specific study.

As stated in the beginning of this section, screening approaches seek the classification of the subject into patient or normal based on the calculation of a subrogated (computer-based) severity measure. Performance validation of these approaches is therefore mostly accomplished by comparing this classification to the equivalent that results from the use of the (manually scored) clinical indices. When this clinical classification is done on the basis of the AHI, the use of a cut-off in thus needed. This cut-off value is indicated in column 8. The associated validation performance measures of the computer method as the results of this dichotomous decision are given in columns 9 to 11. Recall, as stated in Section 3, that these values correspond to the best operation point regarding patient classification (SAHS or normal). Finally, the last column in Table 2 identifies the validation method. Interpretation of the values in column 12 is as follows: “S” stands for “Singleton” which means validation results are obtained from a single dataset of patients (see corresponding references for more details on how this dataset is arranged in each case). “TR/TS” stands for “Training/Testing” and applies to the cases in which the methods have been developed and parameterized using a training set, whereas validation results have been obtained using an independent testing set. It may be the case also where several independent testing sets are used, and in that case this is indicated as “TS1,” “TS2,” and so on. In addition, where necessary, the actual number of patients involved in each of the different sets is indicated in parenthesis. Finally, “CV” means that validation results have been obtained by using a cross-validation process. In those cases, where it applies, also in parenthesis the number of folds used or else “leave-one-out” is indicated.

Analysis of the related bibliography in this context has revealed that computer analysis of the oxygen saturation has been widely used as screening method because of its simplicity and its lower associated cost with respect to the full PSG. In this regard, the most widely used approaches up to the beginnings of the year 2000 relied mostly on the application of different cut-offs over the computed number of oxygen saturation dips (oxygen desaturation) per hour of time, that is, the so-called Oxygen Desaturation Index (ODI). Many other applications could be found also based on the cumulative time spent below certain saturation value (e.g., 90%) [16]. Validity of these methods, however, varies upon the study sample and the selected thresholds. Actually a problem with these approaches has to do with the absence of a consensus to establish the appropriate thresholds.

Bearing that in mind, the work of Daniels et al. [17] describes the system CADOSA which combines analysis of the overnight oximetry with evidence from patients history, a physical examination, and a questionnaire based on the ESS to assess sleep propensity. Fuzzy set theory has been used here, precisely, to account for variability in the definitions and subjectivity of the interpretations. In this work, knowledge representation and description of the inference process is illustrated to represent patients' symptoms and to infer a differential diagnosis with particular emphasis on the detection of OSA, Central Sleep Apnea (CSA), periodic limb movement syndrome, and upper airway resistance [17].

Alternative characterization of SaO_2_ has been tried by several authors by computing an index of signal variability, the delta-index [18–20]. Classical spectral analysis based on Fourier transform has been used to extract relevant features for patient classification based on the power spectrum. Examples of these approaches can be found in the works of Zamarrón et al. [21, 22], later on by Hua and Yu [23], and more recently in Morillo et al. [24]. Among these approximations, in general the method relies on the computation of the Power Spectral Density (PSD) within a specific frequency subband of interest, followed by the application of linear regression analysis to determine the best threshold and assess the predicting performance of the resulting feature.

Based on nonlinear characterization of the saturation signal, it is worth mentioning a first work of Álvarez et al. [25] in which two nonlinear methods, central tendency measure (CTM) and Lempel-Ziv complexity (LZ), are applied to characterize the SaO_2_ recordings. These features are compared against classical desaturation indices, time spent below a saturation of 90%, and delta-index.

Later on, Hornero et al. [27] studied the utility of approximate entropy (ApEn) over the oxygen saturation signal for the diagnosis of SAHS. The study concluded that patients with OSA showed a significant increase in the ApEn values. Similar conclusions were raised by the same authors in another study [28]. In a different work, Álvarez et al. [26] carried out comparison of classical oximetric indices, regularity indices from ApEn analysis, and variability indices from CTM analysis. While results showed that nonlinear methods obtained better accuracy than classical methods, both ApEn and CTM analyses showed very similar discriminative capabilities.

Following the previous hypotheses, subsequent works investigated the use of different machine learning classifiers to help in the classification. Concretely in Victor-Marcos et al. classifiers based on quadratic (QDA) and linear (LDA) discriminants, *k*-Nearest Neighbor (*k*-NN) and logistic regression were tried with different combinations of features including spectral and nonlinear features (ApEn, CTM, and LZ). In this study, the classifier based on LDA with spectral features provided the best performance [29]. In a later study, the authors added a preprocessing step using PCA, improving the previous results of their algorithm [30]. Another relevant work in this context is the one by Álvarez et al. in which a total of 16 features are included in the study and selected by means of a step-forward logistic regression process. The set of features included statistics from both time and frequency domains, conventional spectral characteristics from the power density function, and nonlinear features. Second- and fourth-order statistical moments in the time domain, the relative power in the 0.014–0.033 Hz frequency band, and LZ were automatically selected as the best features [34]. In a recent work, the same authors further investigated the use of genetic algorithms over the same set of features and using a greater sample of subjects. Using a logistic regression classifier, the feature selection process returned as the minimum optimum feature set the one composed of the following six features: first, third, and fourth time statistical moments, median frequency of the spectrum, park amplitude within the 0.014–0.033 Hz, and CTM [35].

Approximations based on the use of ANNs can be found, for instance, in the work of El-Solh et al. [36]. In this work, the authors trained a probabilistic neural network fed with a combination of features to classify patients in either Cheyne-Stokes respiration, OSA, or no sleep disorder. Inputs to the neural network included salient features of frequency analysis, Shannon's entropy, desaturation indices at 2%, 3%, and 4%, and the delta-index. In their experiments, the authors obtained remarkable performance with almost perfect classification of the 213 subjects included in the study [36]. The use of ANNs can also be found in the works of Victor-Marcos et al. [31, 32], in which Multilayer Perceptron (MLP) and Radial Basis Functions (RBF) are used in order to classify the patients as OSAHS or non-OSAHS using nonlinear analysis of the oxygen saturation signal, both obtaining similar results. More recently, and from a regression perspective, Victor-Marcos et al. used a MLP and a linear regression model in order to predict the corresponding AHI for each subject from a set of 14 SaO_2_ features. The MLP model generally achieved higher accuracy in the tested sample. Using different severity thresholds for binary classification, the model improved its performance as the AHI cut-off increases [33].

The previous screening approximations used the oxygen saturation signal as its main source to extract the relevant information. However, besides oxygen saturation, many approaches based on single signal monitoring are used with a similar philosophy for SAHS prediction purposes. In this respect, techniques based on analysis of single-channel airflow recordings constitute another widely extended approximation.

Two recent studies in this respect can be found in works by Gutierrez-Tobal et al. [37, 38]. In the first one, spectral analysis of the airflow recordings is performed and spectral features in the 0.024–0.056 Hz band are combined into a linear regression model. Discriminative capability (patient, control) of the model varied depending on the AHI threshold (5–30) [37]. In the second work, Respiratory Rate Variability (RRV) is measured throughout the signal and spectral and nonlinear features (LZ, ApEn, and CTM) are extracted using both raw and RRV airflow signal. Forward stepwise logistic regression is then used for feature selection and subjects' classification. The highest accuracy was obtained for the model using features extracted from the combination of the raw airflow and RRV signals with 3 out of the 42 features. Only spectral features were included within this set [38].

In the same line of a minimal monitoring requirement, Caseiro et al. [39] propose the application of the Hilbert-Huang decomposition over 5 minutes of oronasal airway pressure signal recorded while the patient is still awake. A similar approach is followed in Salisbury and Sun [40], though using a different index measure as in the approach of Caseiro et al.

### 4.2. Detection of Apneic Events

This category includes those approximations that seek the individual localization of the apneic event in the recording. Thus, whereas estimative approaches described throughout the previous section are oriented toward obtaining a supplementary measure assessing the necessity of the patient to undergo nocturnal PSG, the ones included here rather focus on the direct localization, measurement, and classification of the actual apneic events throughout the recording. An evaluation of the SAHS severity can then be calculated directly by counting the number of events detected and dividing the result by the total sleep time (AHI) or by the processed recording time (RDI) [42].

A summary table of the different event detection approaches analyzed throughout this section is shown in Table 3. Validation results of the different methods are available in Table 4. Table 4 follows a similar structure as described before for screening approaches in Table 2. Thus columns 2 to 5 in Table 4 include study population details, respectively, the database identification, the number and gender of the participants, the age of the population, and the reference severity index. Gold standard for the scoring of the events is again referenced through columns 6 and 7. Similarly to Table 2, for which variability on the hypopnea definition would directly influence the scoring of these events, column 6 in Table 4 contains (when present in the study) a reference to the used hypopnea definition. On the other hand, because evaluation of the detection approach focuses on the capacity to perform individual event localization rather than to perform patient classification, specification of an AHI threshold is no longer needed. Instead column 8 shows the number of events over which detection algorithm is evaluated which have been detected according to the gold standard. Column 9, in addition, states (when possible) the detection precision of the algorithm. In column 9, a “C” means detection is performed in continuum (i.e., limited only by the precision of the signals' sampling rate), and otherwise the number of seconds (step size) that the algorithm uses for each evaluation is shown. Sometimes the algorithm can score events in continuum but validation results are provided using discrete epochs of varying size. These cases are specified using “val” in the corresponding cells of column 9. Sensitivity and specificity and further evaluation measures corresponding to the best operation point of the automatic analysis are provided, respectively, in columns 10, 11, and 12. The last column in Table 4 describes, as in Table 2, the validation procedure. In this case the numbers in parenthesis specify the number of events used for the validation. On the other hand, a “P” in this column stands for “Patient,” meaning that the number of events used in the validation is not specified but the number of patients/recordings instead is.

#### 4.2.1. Analysis of Pulse Oximetry Recordings

The utility of the computerized analysis of the oxygen saturation as screening method for the diagnosis of SAHS has been already mentioned in Section 4.1. Indeed, within the detection setting, a simple strategy to detect and quantify the duration of the apneic event can be very well performed based on the detection of desaturation and/or resaturation patterns in the oxygen saturation signal. The fundamental hypothesis is that the reduction in the respiratory flow caused by each apneic event induces a drop in the arterial blood oxygen concentration levels. Similarly, for resaturation, the assumption is that, after the apneic event causing a respiratory insufficiency, an episode of compensatory hyperventilation must follow, inducing a fast increase in the oxygen saturation levels.

A recent approximation using the oxygen saturation as the source signal is described in the work of Burgos et al. [43] in which detection of the apneic event can be performed in real time. This contrasts most of the approaches in which analysis is usually carried out once the recording has finished (offline). Burgos et al. propose a feature extraction process performed on minute-by-minute time step followed by a classification stage. Several classifiers are tested on the extracted features, the best method being metaclassifier bootstrap aggregating (Bagging) based on alternating decision trees (ADTree). Besides, in this work, the resulting method is afterwards implemented into a mobile PDA device connected via GPRS to the hospital allowing the physician to perform remote patient follow-up [43].

Pulse photoplethysmography (PPG) signal has been proposed as an alternative to traditional arterial oxygen saturation that reflects vasoconstriction changes. In the study of Gil et al., validity of this signal is assessed, and an automatic detector of apneic events based on PPG (DAP detector) is developed and compared to detection methods based on amplitude changes in the airflow and in the oxygen saturation [44].

#### 4.2.2. Analysis of Single-Channel Respiratory Activity

Besides oxygen saturation, detection approximations based on single-channel analysis can be found elsewhere. Using the abdominal breathing signal as reference and focusing on the detection of central events in infants, the work of Macey et al. [45] can be mentioned. In this work, an expert system is designed which relied on the use of ANNs to perform the detection task.

Amplitude based thresholding of different respiratory derivations for online detection of the apneic events is discussed in the work of Reisch et al., including nasal airflow (NAF), thoracic excursion, Pes, and Forced Oscillation Technique (FOT) [46]. Várady et al. also proposed an algorithm for the online detection of apnea and hypopnea events, while their method is based on the analysis of NAF and/or Respiratory Inductance Plethysmography (RIP) derivations. Instantaneous respiration amplitude and interval signals were derived from the respiratory signals and several feedforward neural architectures were investigated. Best reported performance was obtained for the model when using only information from the processing of the NAF derivation [48].

The work of Han et al. describes the development of a detection algorithm based on the mean magnitude of the second derivative of NAF using a thermocouple. According to the authors' results, the proposed algorithm reached better performance as compared with a traditional algorithm based on amplitude analysis [49]. Later on, in Pépin et al. [47], the authors dedicated one channel of an ECG Holter to the recording of nasal pressure and developed an automatic algorithm to search for apneic events in this signal.

The proposal of Nakano et al. is based on the spectral analysis of different airflow derivations [50]. The developed algorithm, based on detection of flow-power dips, was firstly developed using a set of patients in which airflow was measured with a thermal sensor. The resulting method was then evaluated against a conventional time amplitude detection-based algorithm and the detection of oxygen saturation falls (ODI). A second group of patients in which airflow was recorded through nasal-prong pressure transducer and a thermocouple was used to assess generalization capabilities of the algorithm over alternative derivations. Event-by-event validation against visually scored events showed similar behavior for the thermal and for the thermocouple channels; however sensitivity when using these derivations was lower when compared with the nasal pressure sensor. These results agree with the known difference in sensitivity among the tested sensors [73, 74]. In contrast, the algorithm showed stability when using different optimum thresholds across the different channels. With regard to the comparison with other methods, in terms of AUC, the developed algorithm showed better results with respect to other amplitude based analyses of airflow and similar values to the analysis based on ODI [50].

Modeling the diagnostic process but from a particular knowledge engineering perspective is the object of another specific group of methods. Certainly, achieving an interpretable model of intelligent human behavior is the purpose of knowledge-based symbolic approaches. Within this group, the so-called rule-based systems have been widely used for diagnostic tasks in which medical knowledge is coded in form of heuristic rules. Inference in these systems is performed by a process of chaining through rules recursively in which activation depends on the particular case presented at the input [75]. Specifically fuzzy inference systems have demonstrated to be specially suited to model human behavior in domains where data and interpretation processes have a component of imprecision. Medical diagnosis, and SAHS in particular, is a good example of such domains [76].

Following this line, online detection of apneic events is performed using a rule-based system in the work of Shin et al. [51]. The objective is to develop a feedback variable to be used for automated control of CPAP therapy. For that purpose a fuzzy inference system (FIS) is developed using three input variables, and analysis is carried out on a breath-by-breath basis from respiratory airflow measurements: concretely, (1) the relative duration of inspiratory flow limitation in each breath, (2) the degree of hypopnea relative to the past 15 breaths, and (3) the intensity of airflow-derived snoring. This approach contrasts with conventional auto-PAP devices which usually rely on rigid rules based on thresholds over the feedback variable. It allows, in addition, a much greater degree of smoothing in the decision-making process.

Nazeran et al. [52] have developed also a FIS which operates over the airflow variable. The system is designed to differentiate between apnea and hypopnea events, and it used parameters of normalized area and standard deviation. Later on, the works of Morsy and Al-Ashmouny [53] and Al-Ashmouny et al. [54] modeled a sleep apnea detection system using three membership functions to differentiate three regions over the input space of the nasal airflow variable. The authors proposed a two-step approach to classify respiratory events either as abnormal or normal breathing.

#### 4.2.3. Analysis of Multichannel Respiratory Activity

The classical approach for the detection of apneic events in the respiratory signals follows a multichannel approach, and, according to the standards, it involves the analysis of at least one derivation from each of the following signals: airflow, oxygen saturation, and thoracoabdominal respiratory movements [6].

Following this approach, the work of Taha et al. can be firstly mentioned [55]. In this work, the analysis starts with the detection of desaturation events in the oxyhemoglobin saturation signal, and then the sum of RIP is analyzed to detect periods of no breathing. The resulting detected periods are subsequently classified as apneas or hypopneas according to the corresponding baseline breathing reduction. Apneas were further classified into central, mixed, or obstructive based on the presence of abdominal or rib cage breathing efforts [55].

The idea of combining oxygen saturation with other respiration signals has been used in subsequent works. For example, to use the ability of time delayed neural networks (TDNN) to deal with prediction and classification tasks in contexts involving the temporal variable was the main idea of the work of Tian and Liu [56]. In this work, features from the airflow and from the oxygen saturation signals are fed to a TDNN on a second-by-second basis to classify the signal into periods of normal respiration, apnea, or hypopnea. Another example is the work of Sommermeyer et al. [57], in which an oximeter-based PPG signal is used in combination with a nasal cannula for the detection and also differentiation of central and obstructive apneas. For that purpose, pulse wave amplitude (PWA) and pulse rate are calculated from the oximeter recording, and template functions are used for pattern recognition. Respiration effort is derived by analyzing fluctuations of the PWA signal which allow the estimation of an effort ratio.

Steltner et al. developed diagnostic software for the offline detection and classification of respiratory events by analyzing the time series of the nasal mask pressure derivation and FOT, signal as alternatives to the common thoracoabdominal strain gauges and RIP. The method is based on confronting amplitude features from these signals with the appropriate analytically derived thresholds. The algorithm provided results comparable to those of visual analysis by human experts (weighted Kappa statistic) though mean absolute values of agreement were somewhat low (below 0.5) [58].

Almost contemporary, Várady et al. proposed an online analysis approach for both detection and classification of apneic events based on the phase difference between the thoracic and the abdominal respiration [59].

More recently, Houdt et al. [60] published a work in which an algorithm was developed based on breath-to-breath analysis of the respiratory recordings (nasal airway pressure and thoracic and abdominal movements). For that purpose, respiratory signals were divided into half waves using period amplitude analysis and characterized in terms of duration, amplitude, and slope. Apneic events were then detected by dynamical computation of the normal respiratory values and comparing the corresponding respiratory values for each cycle. Further classification of the events into obstructive, central, and mixed was also carried out based on amplitude analysis of the thoracic and abdominal movements [60].

The goal of the study of Al-Angari and Sahakian was to evaluate the classification of whole-night normal and apneic epochs using extracted features from the phase and magnitude of the respiratory effort signals (thoracic and abdominal), compared and combined with some other features from ECG and oxygen saturation signals. SVM classifiers with linear and polynomial kernels were used. The results of their experiment showed that the best performance was achieved when features of the three signals are used [61].

Alternatively, in the work of Waxman et al., the major objective was to focus on the prediction task. For that purpose, their method was tested using different prediction delays and segment durations (30–120 seconds). Several PSG channels were used of which features were extracted using wavelet processing. In their work, the authors proposed the use of a special kind of artificial neural network, namely, LAMSTAR, especially suited to deal with large amounts of data and to provide meaningful relationships to the network's inputs. As expected, prediction performance resulted inversely proportional to the prediction delay. The authors tested also detection on the basis of 30-second segments. For this task they found that while oronasal temperature was the most meaningful signal for the detection of apneas, for hypopnea detection, the most important signal was nasal pressure [62, 63].

Intelligent-based modeling of the respiratory signals (airflow, oxygen saturation, and thoracoabdominal movements) in a multichannel setting has been proposed in the work of Álvarez-Estévez and Moret-Bonillo [64]. In this work signal processing methods are firstly used to extract features over each channel. By using temporal constraint rules, the individual features are then grouped together in form of reasoning units. The resulting reasoning units are finally evaluated by means of a FIS to characterize them as either apnea, hypopnea, or normal breathing with different degrees of membership [64].

The use of fuzzy inference techniques for the detection of apneic events can also be found in other works. For example, based on the previous work of Álvarez-Estévez and Moret-Bonillo [64], Maali and Al-Jumaily have proposed the use of genetic algorithms as a mechanism to generate fuzzy rules directly from data [65]. In a recent work by Otero et al., fuzzy detection methods are also used for marking apneic intervals in the respiratory signals. This method facilitates knowledge acquisition by using an interface to model the membership functions, thus allowing additional system parameterization [66]. In a posterior work, Otero et al. extended the method by incorporating a structural model that permits the projection of a patient's physiological parameters into a computable representation. The purpose of such a representation is to allow the discovery and follow-up of the temporal evolution of different signal patterns of interest [67].

### 4.3. Classification of the Apneic Event Type

The principal goal of the classification task is to characterize the detected apneic event according to the nature of its origin—obstructive, central, or mixed. The main purpose of the works referenced under this is to accomplish such a task.

A summary of the analyzed methods regarding apneic event classification can be found in Table 5. In this table, the third column refers to the possible classes that result as the output of the algorithm. Subsequent Table 6 reports validation results of the different methods. Interpretation of data in this table is similar to that of Table 4 for the detection methods. The fundamental difference lays here in the fact that the classification task does not account for the time localization of the apneic event, but it assumes that the apneic occurrence has been already detected, and the objective is then to classify this occurrence according to its type. Therefore, in Table 6, it does not make sense to specify the classifiable unit in a separate column. And, for the same reason, explicit reference to the definition used for the detection of hypopnea events is no longer relevant. Instead column 7 in Table 6 provides information on the criteria used for the classification of the event type. For the rest, structure of Table 6 is analogous to Table 4.

Among analyzed approximations, several have been already mentioned in the preceding section for which they also included the classification step as part of the algorithm. This is the case of the works of Reisch et al. [46], Taha et al. [55], Sommermeyer et al. [57], Steltner et al. [58], Várady et al. [59], and Houdt et al. [60]. The work of Al-Ashmouny et al. [54] has been previously mentioned as well which extends the single-channel approach described in Morsy and Al-Ashmouny [53] by adding a classification stage using the information extracted from the thoracic and the abdominal excursions.

Besides them, others can be found that center specifically in the classification step. Al-Ani et al. [79], for example, published a method to perform sleep apnea classification using Hidden Markov Models applied to simultaneous recording of NAF, Pes, and Gastric Pressure (Pgas).

Later on, Fontenla-Romero et al. [80] developed a system in which the detection of apneic events is performed from the airflow signal, and, once detected, a wavelet processing is applied to the corresponding intervals of thoracic effort signal. A Bayesian ANN is finally in charge of classifying the interval as central, obstructive, or mixed. The works of Tagluk et al. [81, 82] and Sezgin and Emin Tagluk [78] are also based on the use of wavelets for feature extraction and subsequent classification by means of a neural network. In these works, the airflow signal is examined together with the analysis of the thoracicoabdominal respiration. In this respect, the authors concluded that when features from the thoracic channel are used, better performance can be obtained in the classification.

More recently, Guijarro-Berdiñas et al. have presented a method that combines machine learning techniques and expert knowledge that improves the results of the previous approaches [83]. Maali et al., on their part, have recently proposed a machine learning approach adapting the classical SVMs, resulting in the so-called self-advising SVM technique and applying it to the apnea classification problem [77].

Specifically focused on the classification of hypopneas, in the study of Morgenstern et al. [84], two different methods are presented in order to differentiate between obstructive and central hypopneas. The first technique is proposed using a Pes sensor (an invasive method) in which features are extracted and used to train different machine learning classifiers based on discriminant analysis, SVMs, and Adaboost. The second method was implemented to assess the validity of a noninvasive approach using an airflow tracing recorded with nasal cannula. Similarly, several features were extracted and a diagonal quadratic discriminant was fed with four features. Accuracy of the classification was found lower with the second noninvasive approach [84].

### 4.4. Comprehensive Diagnostic Systems

The term* comprehensive diagnostic systems* is here applied to refer to those systems that comprise—as a minimum—the classification of sleep stages and the analysis of the respiratory activity. This characteristic differentiates comprehensive diagnostic systems from the previous approaches which, in general, are rather specialized in the accomplishment of specific subtasks, such as patient screening, analysis of the respiratory activity, or classification of the apneic event type. In contrast, the approaches referenced in this section involve analysis of the PSG signals in the context of a comprehensive sleep analysis, and thus the name. Ultimately, these approaches are aimed at constituting global solutions in the form of* clinical decision supporting systems* in the context of SAHS.

Table 7 summarizes the works falling under this category and the corresponding validation results are described in Table 8. In the case of comprehensive approaches, the object of the validation may vary depending on the focus of the corresponding reference. Thus, in columns 9, 10, and 11 of Table 8, the word “Events” is indicated when the results refer to detection of apneic events, and otherwise the specific type of the event is indicated in parentheses. On the other hand, when validation data is provided with regard to the capabilities of the method to classify individuals as SAHS patients or normals based on the calculation of the AHI, the code “Diagnosis/X” is specified in which X stands for the AHI cut-off value used for such purpose. Note that this classification is similar to that performed in Section 4.1 when screening approaches were analyzed. The fundamental difference is, of course, the analysis approach. Screening approaches seek the maximum possible simplification of the test, the main objective is to hold a screening function for triage, and, as a consequence, when estimation of the AHI is performed, this is usually done over a simplified subset of the signals contained in the PSG. Analysis in the case of comprehensive systems, in contrast, tries to reproduce as much as possible the full standard analysis of the PSG. In other words, the expected results of a comprehensive system ideally should match the outcome of a full analysis performed by an expert human scorer. Recall nonetheless that, formally speaking, SAHS diagnosis cannot be based exclusively on AHI, but especially when the severity index is low, SAHS diagnosis may require the presence of additional symptomatology [9].

Within this group, we may start by mentioning the system PSG-EXPERT [85], which is presented as the particularization for the case of SAHS of a more general integrated environment for the development of diagnostic expert systems. PSG data are extracted by using signal processing and are inserted into a database organized according to the following categories: clinical history, hypnogram data, sleep parameters, spectral data, EEG time related activity, and non-EEG activity. The system then carries out the diagnosis through a reasoning mechanism over the extracted data which supports the handling of imprecise information using a model of certainty factors [97]. It also includes a validation module which allows testing of concrete patient's cases by comparing the results of the analysis with those of the medical experts. Perhaps the main limitation of the system is due to its general purpose philosophy, for which it just operates at the symbolic level. In other words, processing and segmentation of the raw signal have to be performed separately and the resulting data must be afterwards inserted into the database. In spite of its built-in validation module, no validation results have been reported assessing the actual performance of the system [85].

Following with the symbolic perspective, Ugon et al. [86] present an approach that relies on the fusion of complex symbolic objects connected to each other following a set of simple predefined rules. The approach of Ugon et al. incorporates a module that handles sleep scoring following a signal processing approach, which extracts features from the neurophysiological signals, and uses binary decision trees to end up with the final labeling of the sleep epochs. Graphical but not numerical validation results of this system can be found in [86].

The system TASAS emerged from the particularization for SAHS of a more general framework for the design of expert systems with special emphasis on the handling of temporal knowledge [87]. Concretely, the system serves from the use of the Causal Constraint Temporal Networks (CTCN) representational model which allows handling of temporal information between symbolic items, modeling them either as points or as intervals, and implementing effective mechanisms to manage causality [98]. Under the modeling framework provided by TASAS, expert knowledge can be implemented describing temporal patterns that are used to detect physiological evidences of apneic events in the PSG. Validation details of the TASAS system can be found in [87]. A recent extension of this model to support uncertainty in the reasoning process by means of the fuzzy logic paradigm can be found in Fernández-Leal et al. [88].

Two important considerations have to be taken into account at this point. First, while the three previous systems carry out the diagnosis from a knowledge-based perspective, these systems actually lack built-in procedures for the analysis of the raw signals. They instead operate at the symbolic level. Second, modeling of the causal and temporal relationships between the different events requires the so-called knowledge acquisition stage to make medical expertise explicit in the form of temporal and/or production rules. However, this is a well-known bottleneck in the design of expert systems [99].

The approximation of Guimaraes et al. tries to face the previous shortages and presents a method for the discovery of temporal patterns in multivariate time series and their subsequent conversion into a linguistic knowledge representation. The method is specifically applied to the context of SAHS diagnosis [89]. The idea relies on the use of several levels of abstraction on a bottom-up analysis schema. In this respect, self-organizing neural networks are used to discover elementary patterns in the time series, and machine learning algorithms are subsequently used to process the patterns generating a rule-based description. At the next levels, temporal grammatical rules are inferred. Although the work of Guimaraes et al. cannot be regarded as a complete comprehensive approach, for which it only involves the set of respiratory signals, it does constitute an interesting integrative framework by the linking of several artificial intelligence methods and face the problem of knowledge acquisition on multichannel PSG time series. Further evaluation of the method has to be performed though, as the authors themselves suggest, in order to assess its actual clinical relevance on the context of SAHS diagnosis [89].

The system SAMOA is one of the first approaches to integrate both artificial intelligence and classical signal analysis techniques for the development of an integrated product which, in addition, is able to provide explanation of its results [90, 91]. Architecture of the SAMOA system is mainly integrated by four different modules: (i) the polysomnographic prescription module, (ii) the module for the characterization of the respiratory activity, (iii) the module for the construction of the hypnogram, and (iv) the diagnostic module.

While the system SAMOA solves many of the problems of its predecessors, still a common drawback on these systems is the use of fixed protocols and thresholds while automatically analyzing the raw signals from the PSG. For example, the use of fixed thresholds to identify amplitude changes in the respiratory cycles may cause incorrect assessment of the baseline breathing. On the other hand, still in SAMOA, besides counting with mechanisms for generation of the hypnogram, analysis of the raw EEG for the detection of the different rhythms and transitory components is carried out by using a supplementary system [100].

In an endeavor to solve the previous drawbacks, the MIASOFT system has been recently developed [92]. This system contributes to supporting its analysis capabilities under two fundamental pillars: (i) its comprehensive approach, in which neurophysiological activity is used as a context for the interpretation of the detected respiratory events, and (ii) the implementation of mechanisms to handle data imprecision which mimic human's reasoning procedures under the principles of generalization and approximation [92]. There are two well differentiated groups of modules in the MIASOFT system: (i) those specialized in the analysis of the respiratory activity and (ii) those specialized in the processing of neurophysiological activity. Respiratory analysis is structured into three submodules for the identification of apneic intervals, characterization of the SaO_2_ signal, and the analysis of the respiratory effort [93]. On the other hand, neurophysiological analysis is organized into seven submodules: three are responsible for cerebral activity characterization, eye movements' detection, and muscle tone analysis. This neurophysiological information is fed to a module in charge of obtaining patient's hypnogram [94]. MIASOFT system, besides, is provided with mechanisms for the analysis of the sleep microstructure and to deal with the detection of transient events including microarousals [95, 96], sleep spindles, and K-complexes. The whole analysis in this system is concurrently assisted by additional modules providing functionality for artifact detection, temporal information correlation, and inference with special emphasis in the support of uncertainty and imprecision in the decision process [92].

## 5. Analysis and Validation of Commercial Approaches

Even though current automatic SAHS diagnostic systems still present some shortages, for the clinician, the simple fact of being able to carry out an offline analysis over a digitalized recording yet represents an important evolution. Advantages include not only the amount of saved paper, but many others such as the possibility to visualize the signals over different time and amplitude scales, easiness in the annotation of the detected events, or the possibility of incorporating supporting tools that automate, at least in part, the scoring task. Indeed, from a commercial perspective, there is already some time since the sleep labs were equipped with computer scoring systems that operate over the fully digital overnight registrations.

An example is the system Polyman, which strictly speaking is not commercial software, but it is an EDF(+)-compatible viewer and sleep scoring supporting program originally created by Kemp and Roessen [101]. Polyman includes several aiding tools that can perform automatic analysis over the common sleep scoring subtasks. The program allows the user to set up diverse configurations for visual analysis of the digital recording. Each signal can be freely filtered, adjusted, and automatically analyzed. Automatic analysis of frequency content (FFT), threshold crossings, neuronal feedback analysis [102], and rectified EMG can be applied to the signals on the screen. It also supports manual scoring of sleep stages, apneas, leg movements, and arousals according to standard R&K or AASM rules. The scorings are kept in standard EDF+ files and a report of standardized sleep quality parameters can be produced. A licensed version is also available that includes additional modules for the automatic scoring of limb movements, respiration, body position, pulse rate, ECG, and oxygen saturation. Polyman does also support synchronization with video files [103].

The trend is indeed during the last years in which new commercial systems are continuously appearing in the market providing new capabilities regarding automatic analysis of the PSG. The ultimate use and acceptation of these automatic capabilities in the clinical routine, however, are still under discussion, for which further and proper validation of these systems is of fundamental importance. In the following we shall briefly discuss some of the validation studies that have appeared during the last years on the research literature regarding commercial approaches that perform automatic analysis in the context of SAHS.

Table 9 summarizes the corresponding literature review on this topic. In Table 9, the second column makes reference to the device type according to the commonly used classification proposed in 1994 by the American Sleep Disorders Association [104]. According to this classification, Type-1 category refers to the standard clinical PSG, considered as the reference to which the other monitor types are compared. Type-2 devices incorporate a minimum of seven channels, including EEG, EMG, and EOG, allowing sleep staging, and a set of respiratory signals, at least ECG, airflow, respiratory effort, and oxygen saturation, therefore allowing calculation of the AHI. Type-3 monitors incorporate a minimum of four channels with at least two channels of respiratory movement, or respiratory movement and airflow, and usually heart rate and oxygen saturation. Direct estimation of the sleep profile (hypnogram) with Type-3 devices cannot therefore be performed and only estimative subrogates of the actual AHI (e.g., RDI) can be obtained to evaluate the SAHS severity. Finally, Type-4 monitors use at least one respiratory channel, usually either airflow or oxygen saturation. A monitor that does not meet the criteria for Type-3 (i.e., a monitor that measured one to three channels or does not include airflow despite having four channels) is classified as Type-4 [104].

Columns 3 to 6 in Table 9 include study population details, respectively, identification of the dataset, number and gender of the participants, age of the population, and the reference severity index. Gold standard for validation is defined in columns 7 and 8. In column 8, explicit reference to the definition used for the scoring of hypopnea events is given when available for the corresponding study. The reason for that is that validation studies of this kind of devices do usually consider the AHI reported by human expert scorers as its reference, and as it was the case before for screening and detection approaches (see Tables 2 and 4), this index can be partly conditioned by the variability associated with the definition for the scoring of these events. Column 9 of Table 9, in addition, indicates whether the recordings analyzed by the computer method were carried out in in-lab attended conditions (L) or were performed at home (H). Gold standard reference AHI cut-off value used for the validation of the system for patient classification is shown in column 10. The corresponding quantitative validation measures of sensitivity and specificity are shown in columns 11 and 12, and additional validation measures such as the area under ROC curve (AUC), Kappa index (*κ*), intraclass correlation coefficient (ICC), and Pearson's linear correlation index (*r*) are shown in subsequent column 13. Finally a difference with previous validation tables in previous sections is that, in Table 9, the column showing the validation method is omitted for simplification given that in all references the method used is “S.”

A first example of validation of Type-1 devices can be found in the work of Pittman et al. [105] which describes the validation of the Morpheus I (WideMed Ltd., Israel) sleep scoring system. This system is aimed at performing a comprehensive analysis of the PSG recording, thus including both neurophysiological and respiration data.

Somnolyzer 24 × 7 (Philips Respironics, The Netherlands) is also comprehensive software that has been integrated into an e-Health solution platform. It works as a centralized server so that involved centers can upload their resulting PSGs, receiving back scorings and the corresponding report. Description of the system and validation results according to reliability in automatic sleep scoring capabilities regarding R&K criteria [135] can be found in Anderer et al. [136]. Posterior adaptation and validation regarding the AASM criteria [137] have been published in Anderer et al. [138]. Regarding analysis of respiratory signals and SAHS diagnosis, Woertz et al. [107] described the use of this system to test a database of 51 subjects and assess the AHI agreement in comparison to human scorers.

Validation of the *α*-Somnostar 4100 (SensorMedics Corporation, USA) system has been performed in the work of Barreiro et al. [108]. This system records signals of EEG, EOG, EMG, ECG, digital pulse oximetry, thoracic and abdominal movements, body position, and oronasal flow. The software provides functionality for the automatic analysis of the respiratory function and for the classification of sleep stages from the EEG. According to the validation of Barreiro et al., however, low concordance has been shown between results of the automatic analysis and those of expert's manual revision, especially in the detection of hypopnea events and in the classification of sleep stages [108].

### 5.1. Portable Devices

Parallel to the development of in-lab systems, a growing interest in the last years is toward the design of portable devices for the outpatient (in-home) monitoring. In general, literature regarding validation of portable devices in the diagnosis of SAHS is extensive and previous reviews in this respect can be found in [104, 139, 140] and more recently in [42] and in [141]. Still today, however, the actual role of portable devices as to substitute full PSG in the diagnosis of SAHS has to be properly assessed.

Focusing on automatic analysis, Table 9 shows validation results of some of these commercial portable monitors. In this respect, and as it was previously stated, we may specifically consider as portable the devices under categories Type-2, Type-3, and Type-4 [104].

Morpheus Hx (WideMed Ltd., Israel) is a bedside computerized analysis system that can be connected to a standard hospital monitor and utilizes mainly respiration (respiratory impedance, end-tidal carbon dioxide, and SpO_2_) and ECG, for the extraction of autonomic nervous system activities yielding to sleep and wake states, and detects respiratory events using the morphology of the respiration and desaturation [109].

Apnea Risk Evaluation System (ARES, Advanced Brain Monitoring Inc., USA) is a Type-3 device composed of a hardware part that carries out the signal acquisition, ARES Unicoder, and software performing analysis of the recorded information, ARES Insight software [110]. The device records the following signals by means of a casing attached to the patient's head: oxygen saturation, pulse, snore (through a microphone), and body and head position (using accelerometers). In the validation study of Ayappa et al. [111], the system is expanded by adding measurement of airflow by nasal cannula connected to a pressure transducer. From one recording, ARES Insight software analyzes the signals to compute an indirect measure of the number of apneic events per hour of recording (RDI). Calculation of this measure is based on the analysis of the oxygen saturation signal searching for desaturation and resaturation patterns to obtain an estimation of the number of apneic events. Incorporation of airflow measure also allows computation of AHI based on the airflow channel. Measures comprising pulse, snore, and sleep position serve as contextual information to discard invalid desaturation and also to detect awakenings that confirm the presence of the apneic event. These awakenings should not be confused with EEG arousals since the software does not provide any analysis capabilities over the neurophysiological signals. Validation results of this system can be found in the works of Westbrook et al. [110], Ayappa et al. [111], and To et al. [112].

Sommocheck (Weinmann GmbH, Germany) is Type-3 device addressing portable monitoring of patients in risk of SAHOS. It consists of a device attached to the chest of the patient that records the signals of airflow (thermistor), snore (microphone), oxygen saturation and pulse (finger sensor), and body position (using an integrated sensor in the main unit) [113, 142]. Software provided with the unit allows the physician visualization and automatic analysis of the recorded signals. The analysis counts the number of desaturation and apneic events (apneas and hypopneas) and calculates basic parameters of ODI, Apnea Index (ApI), Hypopnea Index (HI), and AHI, respectively, as the number of desaturation events, apneas, hypopneas, and apneas/hypopneas per hour of sleep. In the validation study of Ficker et al. [113], automatic analysis capabilities of these devices on in-lab PSGs are tested against experts manual scoring.

Type-4 WristOx 3100 (Nonin Medical Inc., USA) consists of two parts: pulse oximeter hardware (Nonin WristOx 3100) and software for the analysis of the recorded SaO_2_ signal (nVision 5.0) [114]. SaO_2_ signal can be recorded at several sampling frequencies (1 Hz, 0.5 Hz, and 0.25 Hz). The analysis algorithm is based on the counting of the number of desaturation events to estimate the amount of apneic occurrences per hour of sleep. Validation results for this device can be found in the work of Nigro et al. [114].

WatchPAT (Itamar Medical Ltd., Israel) is a Type-4 device worn around the wrist with two finger probes that extend from the main body of the device; one is an opticopneumatic sensor that carries on registration of the Peripheral Arterial Tonometer (PAT) signal and the other measures arterial oxygen saturation [115]. Heart rate signal is derived from PAT and the body of the device also contains an actigraph to estimate sleep time and differentiate it from wake time [115, 116]. Respiratory events in this device are detected with an automated algorithm using a combination of PAT signal attenuation, desaturation on pulse oximetry, and changes in heart rate [116, 143]. For validation details on this device, the reader may want to check the works of Ayas et al. [115], Bar et al. [116], Pittman et al. [117], or Zou et al. [119].

ApneaLink (ResMed, USA) is a Type-4 three-channel device that measures airflow (nasal pressure transducer), oximetry, and pulse, providing an AHI estimation based on recording time. Both automated and hand-scored methods are available through the ApneaLink software. In the automatic setting, by default an apnea is defined as a decrease in airflow by 80% of baseline for at least 10 s with maximum duration of 80 s. Hypopnea, on the other hand, is defined as a decrease in airflow by 50% of baseline for at least 10 s and with maximum duration of 100 s [120]. The ApneaLink does not distinguish between obstructive and central events because the signal is based only on airflow and there is no recording of respiratory effort [121]. The sampling rate of the flow signal is 100 Hz, with a flow-sensor effective range of −10 to 10 cmH_2_O and a 16-bit signal processor. The signal is processed by linearizing, filtering the noise and zeroing to baseline. Flow measurements are digitalized and downloaded to a PC [122]. Validation of the device has extensively been done and in the literature several references can be found in this respect [120–125].

Somnolter (Nomics, Belgium) type-3 device records nasal flow, oxygen saturation, body position, thoracic movements, and jaw movements. The automatic analysis of the traces is based on a multisignal approach to detect sleep apneas/hypopneas and sleep/wake states [126]. For the recording of the midsagittal jaw movements, the voltage is sampled at 10 Hz, digitally linearized, and the corresponding mouth opening is stored with the other PSG channels in an EDF file [144]. The automatic scoring applied over the nasal airflow to delineate respiratory events of at least 10 seconds is then based on the following rules: apneas occur if an airflow reduction of more than 80% appeared within 10 seconds; hypopneas are scored if an airflow reduction of more than 30% occurred in association with desaturation of ≥4% or with a salient jaw movement following the respiratory event, connected with an arousal [126]. A salient jaw movement is characterized by high amplitude or discontinuous movements in the time signal. The system is also able to classify apneic events into obstructive, central, and mixed [145]. With regard to the sleep/wake trace provided by the automated analysis, wake states are characterized by “up” body positions or very high or “chaotic” jaw activity [146]. Validation of automatic scoring performance against simultaneous visual-scored in-lab PSG recordings has been carried out in Cheliout-Heraut et al. [126].

Embletta device (Natus Medical Inc., USA) is a Type-3 monitor that consists of a nasal pressure detector recording the square root of pressure as an index of flow, thoracoabdominal movement detection through two piezoelectric belts, finger pulse oximeter, and body position detection. A comparative study against in-lab PSG using this device has been performed in Dingli et al. [127] and in Ng et al. [128].

The Lifeshirt (Vivonoetics, USA) is a form-fitting vest that comes with sensors embedded within which are capable of monitoring a range of physiological parameters including respiration via RIP, heart rate, oxygen saturation, and motor activity [147, 148]. Thus it can be classified within the Type-3 category of portable devices. Analysis of ventilation through RIP bands in the Lifeshirt is performed through a propriety algorithm that computes breath volume and compares it to the median breath volume of the preceding two-minute interval. In the article published by Goodrich and Orr [129], validation of autoscoring capabilities of the device was compared against manual scoring of PSG. Manual editing of the automatically generated results was allowed so that when there was any discrepancy between the automated analysis and the technician (e.g., due to artifact), the technician could rescore part or all of the sleep study to make it more accurate. Validation study of this device can also be found in the work of Carter et al. [130].

The RUSleeping RTS (Philips Respironics, The Netherlands) is a Type-4 screening device with a single channel that monitors changes in nasal pressure with a nasal cannula, pressure transducer, and recording unit that provides signal processing and data analysis [133]. The device monitors changes in nasal pressure to detect respiratory events and it calculates an index representing the number of these events per hour of the overnight recording in real time [133]. More details about the automatic scoring and the corresponding validation results can be found in the work of Grover and Pittman [133].

BreasSC20 (Breas Medical AB, Sweden) Type-3 system consists of a four-channel device in which oxygen saturation is measured by a flex sensor, and airflow is measured after the square root transformation of the recorded nasal pressure using a nasal cannula. From the nasal pressure, in addition, the snoring signal is extracted. Thoracic and abdominal movements in this device are measured independently by two bands with a piezoelectric crystal, which also keep track of the body position [131]. Once the recording has been done, the data is downloaded to PC software where the Breas Analysis Software would provide indication of the presence of SAHS and other associated disorders. For more details, the reader is referred to the validation studies of Núñez et al. [132] and Ruiz-López et al. [131].

The Stardust II (Philips Respironics, The Netherlands) is a Type-3 portable monitor designed to measure and record five diagnostic parameters: SpO_2_ (via finger probe), pulse rate (from the oximeter probe), airflow (pressure based airflow through a nasal cannula), respiratory effort (piezoelectric sensor in a belt placed midthorax), and body position (built-in mercury switch) [149, 150]. Data collected is stored on internal memory in the device and then downloaded to a computer for automated analysis by the host software (Stardust Host Software, Respironics, Inc., USA). Yin et al. [134] evaluated the automatic scoring algorithm of this device, however finding low agreement and suggesting that data analysis should be performed manually.

## 6. Discussion

Throughout the previous sections the state of the art has been reviewed comprising computer methods for the automatic diagnosis of SAHS. The growing interest in the field of sleep medicine, together with the recent advances in computer analysis methods, have contributed to the increment of the number of developments in the field in the last years. The heterogeneity of the different techniques and the increment of the scientific production make it somewhat difficult to follow the trace of the recent developments in the area. This situation motivates a literature review to be performed whose objective is to serve as a reference for the reader interested in the development of computerized methods for the diagnosis of SAHS. Specifically, the authors have focused on the analysis of the research literature over the last 15 years.

A remark has to be done regarding the meaning of the word diagnosis in the context of this review. In this respect, diagnosis has to be rather interpreted as the capacity of the computer method to intercept a well-defined severity of AHI when the automatic analysis of the PSG is done. Indeed it has to be taken into account that, according to the last version of the AASM manual for the International Classification of Sleep Disorders, formal diagnosis cannot be based exclusively on the AHI, but, depending on the resulting severity, diagnosis of SAHS may require the AHI to be accompanied by additional symptomatology [9].

Taking the previous into account, analysis of the bibliography has effectively shown how the referred context yet constitutes a field with a certain maturity, despite the relative youth of the sleep science, where the number of approximations has significantly increased, especially in the last years. Even if the scope of the review has centered on automatic methods that operate over the mostly extended signals used in the clinical routine (see section VIII of the AASM manual [6]) the resulting number of approaches is noticeable. Actually, the reality is that such methods are yet bypassing the research frontiers, and several solutions are already present in the market (see Section 5). Acceptation of these systems as well as their actual use in the real practice still remains, however, somewhat low among the medical specialists. It can be said, in fact, that implantation of the computer-based SAHS diagnostic systems in the clinical routine is still at a preliminary stage.

Complexity of the analysis task is undoubtedly playing a role for this slow transfer. This complexity can be reflected in the fact that many of the current available tools still nowadays only partially fulfill some of the required subtasks that integrate the whole diagnostic process. For example, there exist SAHS diagnostic systems providing capabilities for the analysis of the respiratory activity, which nevertheless present deficiencies from the neurophysiological point of view, and vice versa. In this review, this is noticeable, for example, when examining the number of comprehensive approaches in comparison with the rest of the methods.

Two concurrent trends may be differentiated by examining the previous literature review. On the one hand, the first trend is focused toward reducing the required montage, translating the complex full monitoring carried out in the attended hospital environment, to a more lightweight setting which can be ultimately performed in unattended conditions, even at the patient's home. On the other hand, once the recording has been done, and especially motivated by the recent advances in the field of signal processing and artificial intelligence, new analyzing algorithms are breaking through with the purpose of automating the analysis of the resulting data.

Certainly, and although somehow related, these two trends can be considered separately. While reducing the number of signals to be monitored has several advantages, including reduction of costs, more comfort for the patient, or reduction of waiting lists (more recordings can be scheduled in parallel without being constrained by the number of beds in the hospital), still manual scoring of the resulting data is complex and time-consuming. For this purpose, automation of the scoring process is still needed.

Throughout the analysis performed in Sections 4 and 5, Tables 1 to 9 are intended to serve as a quick reference summarizing the different covered methods. In particular, Tables 2, 4, 6, 8, and 9 are meant to hold validation details of the different approaches for reference and comparison purposes. Comparison of the results from one method to another, however, is not always straightforward. First, it is well known that usually validation results are heavily dependent on the used database (demographics of the population, number of subjects, balance of the classes, etc.) and on the design of the validation tests. Some of these details are included within the mentioned tables; however complete coverage of all influencing factors would be unattainable. Besides, it is worth recalling that values of sensitivity and specificity reported in those tables (especially in Tables 2 and 9 where the validation results have strong dependency on the selected AHI threshold) correspond always to the best operation point of the method when using the automatic analysis. Results in Tables 2, 4, 6, 8, and 9 can be therefore regarded as optimistic and more in-depth analysis would reveal that, in many cases, performance results are extremely dependent on the experimental conditions. Variability in the recording conditions, in the scoring criteria, or in the validation metrics, makes it difficult to carry out objective meaningful comparisons among the different methods. The opinion of these authors is that validation results contained in these tables should be considered for guidance only and that they have to be carefully taken into account. The interested reader is aimed at checking within each of the provided references for more details.

Such a situation evidences the need to further standardize the validation process in order to achieve more meaningful comparisons among the different methods. A standardization process like this would encourage the use of open and consensus databases for which standard tests would be designed, probably organized into different tasks. Each task would fulfill specific necessities demanded from the clinical practice, and, for each one, the proper validation process to assess its degree of accomplishment would also be standardized. Such a procedure, besides allowing more objective method comparison, would set concrete objectives and would help guide the development of future automatic analysis methods, filling the gap between engineering research and clinical necessities.

It is nonetheless undeniable that recent advances in computer technology are already contributing and opening new possibilities in the diagnosis of SAHS. Rapid screening of risk subjects is, for example, the main objective of the methods analyzed throughout Section 4.1. Most of the approaches analyzed in this respect (see Table 1) rely on the analysis of a single channel, either by the monitoring of the oxygen saturation or through some derived measure of airflow. With respect to the first group (analysis of oxygen saturation), results from Table 2 can be compared to those of a previous review of Netzer et al. in 2001 [16] which reported screening sensitivity and specificity varying, respectively, between 41–100 and 31–98 for AHI cut-offs in the range of 10–15. In the more recent review of Maurer [151], using similar thresholds (5–15) screening sensitivities for these kinds of methods ranged between 65–100 with specificities between 23–92. Looking at Table 2, our analysis reveals sensitivities between 78–100 and specificities between 54.2–100 (76.7–100 and 70.8–100, resp., regarding methods using airflow). These values, which have to be carefully interpreted for the reasons already discussed, seem to nevertheless confirm a previously reported trend in the literature (see also [151–156]) for which, in general, screening methods for SAHS diagnosis tend to be biased toward sensitivity.

Assessing validity of screening approaches, however, is not an easy task, and in the case of OSAS some researchers are raising the voice, for example, on the importance of evaluating the results in the context of the prevalence of the disease [157]. An additional problem to evaluate screening approaches comes by the extended use of thresholds. Indeed the problem has to do precisely with the absence of a consensus to establish the appropriate limit value, causing validity of these methods to differ upon the study sample and the selected cut-off.

In contrast to screening and estimative approaches, detection approaches analyzed in Section 4.2 (see also Tables 3 and 4) involve the direct assessment of the associated severity for which they seek individual detection of each single apneic occurrence. Among them, some approaches can be found that rely as well on the analysis of the oxygen saturation signal. Indeed, because of its simplicity, relative robustness, and easiness of registration, early there can be found several computer approaches attempting apneic event localization on the basis of this signal. Besides the methods analyzed here, the works of Netzer et al. [16] and Flemons et al. [42] include a good review of methods prior to the year 2000. A general limitation with the algorithms resulting from the processing of the oxygen saturation is, however, the incapacity to distinguish between apneas and hypopneas or to carry out a classification of the detected event as obstructive, central, or mixed.

Approaches carrying out the analysis of different respiratory channels such as nasal airflow, thoracoabdominal respiratory movements, Pes, or FOT have been described as well. These approaches may involve more complicated techniques, but, as a result, more information about the nature of the apneic event can be extracted. Within this group, approaches based on the analysis of single respiratory channels are usually more prone to suffer from external artifacts, especially those originated from the movement of the patient. In general, more interesting approaches can be obtained when the algorithm is implemented over a multichannel environment, gathering information from several cardiorespiratory derivations. These approximations require dealing with the time correlation of the individual events along the different channels, which complicates the algorithm. On the contrary, multichannel approaches usually lead to a more accurate detection of the apneic event and its posterior classification. In particular, results in Table 4 show that detection of apneas is relatively easier when compared to hypopneas. This is not surprising for which an apnea represents an event of bigger intensity which is easier to identify in the PSG. Hypopneas, on the other hand, are more subtle events, and even more the standard definition for this kind of events has been historically a matter of discussion [5, 41]. An excessive dependence on the use of thresholds produces again negative consequences in the detection context. For example, a common problem for the analysis of the respiratory activity is the establishment of the normal respiration baseline. In this context, the use of fixed thresholds to identify amplitude changes in the respiratory cycles may cause incorrect detection of several apneic event occurrences. This is of special importance in cases with subsequent repeated occurrences of airflow reductions—as it is common in severe SAHS patients—and for the correct detection of hypopnea events.

Dependency on the appropriate setting of thresholds or critical values is indeed an issue shared by many of the approaches analyzed in this review. It is a difficult question, almost philosophical, because part of the problem actually has to do also with the different approach followed by a computer program and the human scorer when facing the resolution of a decision problem. Indeed computer programs usually carry out a discriminant analysis partitioning the output space into disjoint sets (e.g.,* yes* or* no*) leading to categorical decisions and classifications. Human decision-making processes, in contrast, usually serve the use of generalization and approximation and are affected by uncertainty. Sleep diagnosis is not an exception, and imprecision is inherent to the domain and comes out of different sources. The presence of noise in the signals, data dependency, redundancy, limited sensitivity of the transducer, loss of information due to the analog-to-digital conversion, interferences, or expert's intra- and intervariability are some examples. Moreover, it is usual within the clinical language that opinions are given in terms of possibilities rather than in certainties, possibilities over which qualitative terms are used in contraposition to the use of quantitative terms (and thus in contraposition to the use of fixed numeric thresholds). Given this context, the necessity of building systems emerges that are able to handle heuristic knowledge and vague expressions and to carry out the decision-making from a qualitative point of view. Recent developments in artificial intelligence and signal analysis may play an important role and become part of the solution to this problem.

Classification of the detected events as obstructive, central, or mixed represents a further step in the diagnosis and an additional challenge for the automatic scoring program. This review has analyzed several methods in this respect which are summarized in Tables 5 and 6. According to results in Table 6, it seems that discrimination of mixed events represents the most difficult task. Only in the work of Reisch et al. [46], this class has been the most accurately detected. However, this holds only when either the Pes (which is invasive) or the FOT signals are used, both of them rarely used in the clinical practice. In addition, it has to be considered that the definition of mixed events is also controversial for manual scoring [158] and thus scoring of these events is expected to be specially affected by interscorer variability.

Comprehensive approaches represent an alternative view. Methods analyzed within this category do not involve the simplification of the monitoring montage. In contrast to previous approaches, it is their aim to help the clinician, saving time and reducing the complexity of the PSG analysis, however, while respecting the full standard scoring procedures as much as possible.

Development of comprehensive approaches therefore represents a great challenge from the perspective of software engineering. Computer analysis of a full-night PSG involves a considerable amount of data to be processed, in which information has to be meaningfully correlated in time among the different channels to detect patterns of clinical relevance. The processing of several PSG channels implies the application of different analysis methods in order to extract the respective events of interest. Artifacts and interactions among the whole set of derivations have to be carefully taken into account. Moreover, for a system to be regarded as a supporting tool to aid in clinical decision, it is desirable that it provides interpretable outputs. The previous necessarily involves accepted medical knowledge to be implemented in form of heuristic rules, which make knowledge of the system explicitly available to the physician. Tracing of the reasoning processes and follow-up of the diagnostic procedure should be enabled in the system.

In this respect, and from a software engineering perspective, it seems that while signal processing methods are more useful to extract the relevant information from the patient's biosignals, artificial intelligence techniques are more suitable for the analysis and interpretation of the extracted features. Specifically, for the analysis and interpretation of the symbolic events, knowledge-intensive approaches are preferred in contraposition to data-driven approximations. Effectively, although higher precision can be achieved with the use of data-intensive approaches such as SVMs or neural networks, a problem is the absence of any explanation capabilities of the results. Indeed, from the user perspective, these methods ultimately behave as black-boxes.

A survey of validation studies regarding commercial approaches has been performed throughout Section 5 and summary results are contained in Table 9. The analysis reflected the growing interest toward the design of portable devices for outpatient (in-home) monitoring. Out of 28 studies, only 3 referred to validation of Type-1 devices, while, from the remaining 25, 13 dealt with validation of Type-3 devices and 12 of Type-4 devices. However, from the same 25 regarding portable devices, only 9 did actually contain data from in-home monitoring. Of them, 6 performed a comparison between different-night in-home and Type-1 in-lab registrations, 1 used same-night Type-2 monitoring as reference, and 2 used same-night Type-3 registrations.

Sensitivities and specificities on the 6 studies with different-night comparison of in-home monitoring with Type-1 registrations varied between 92–100 and 50–100, respectively, in the case of in-lab conditions and between 79.4–100 and 76.5–100 in the case of home monitoring. Thus, although common sense dictates that in-home registration may introduce additional sources of noise and unreliability in the automatic scoring, it is difficult to extract such conclusions from the results of Table 9. It has be beard in mind, though, that these results are expected to be affected at least by internight and interstudy variability. Results are not conclusive when we eliminate the interstudy factor and we look at the individual results for each study. On the other hand, some studies in literature have also suggested that reliability of in-home recordings, when manual scoring is performed by trained technicians, is similar to that achieved on attended in-lab conditions [159, 160]. Unfortunately, none of the studies have tried to make a comparison between same-night in-lab registration and same-night in-home registration using the same system. Such a study would have shown interesting data on whether the recording conditions could have a true significant influence on the performance of the automatic scoring.

Nonetheless, and although portable devices may not offer the full functionality of the comprehensive approaches, they have the great advantage of allowing the patient to be monitored at home. A screening diagnosis can then be issued, serving as first alert or control, and be used for triage reducing dependence from the hospital resources. That means reducing of waiting times, reducing costs, and increasing the number of diagnoses. The reduced cost of in-home studies in comparison to those carried out at the hospital has been even reported in a study by Golpe et al. [161] under the condition that a supporting technician would be required to attend to patient's home. Usually, however, this is not even necessary since the patient can be prepared for home monitoring at the sleeping center by trained staff. The trend is in fact toward the increment of portable monitoring, a practice with an increasing role (although not yet decisive [42, 162]), with growing support from scientific societies, and is already included in the clinical routine of many sleep centers worldwide.

Integration of portable devices into an intelligent telemonitoring platform would be the next step, according to the authors, in the development and expansion of portable sleep monitoring. Such a platform would allow the medical staff to increase the monitoring by remote follow-up of the patient. Indeed, due to the high costs associated with the in-hospital PSG, current diagnosis is usually constrained to data from limited, if not just only one-night, registration. This represents a major limitation for current sleep studies which may bias the diagnosis, especially in the cases where the disease does not manifest every night (e.g., as in the case of some sleep parasomnias). Discussion is over the table in the medical field about second-night effects, or whether monitoring of the patient out of his/her normal sleeping environment—as done in in-hospital night monitoring—accurately reflects or can instead influence the actual sleeping pattern. It seems reasonable to believe, though, that an increment in the number of monitored nights would contribute to getting rid of the one-night bias effect and help to come up with a better diagnosis. Integration of portable devices into an intelligent telemonitoring platform would permit, in this respect, lowering the cost and allow more periodic patient monitoring to be performed. The term intelligent is important, however, because increased monitoring ultimately means increased amount of data to be analyzed, and therefore eventually some kind of automatic analysis must be introduced in the process for it to be scalable.

## 7. Conclusions

The number of contributions regarding computer-assisted diagnosis of SAHS within the last 15 years is prominent. Despite the relative youth of the sleep medicine, analysis of the state of the art has effectively shown how the referred context yet constitutes a field with a certain maturity. Several methods have been analyzed which focus on different subtasks of the diagnostic process, from the screening and early diagnosis to full comprehensive systems. Indeed, nowadays computer-assisted SAHS diagnostic systems are already bypassing the researching frontiers with several solutions currently present in the market. The actual use of the full automatic scoring possibilities of these systems in real practice, however, still remains low.

Current efforts in the design of these systems are focusing on the design of robust algorithms which can be used to accurately interpret data registered in ambulatory conditions. This should contribute to unlocking the bottleneck of the centralized in-hospital diagnosis and reduce the costs associated with the test. Development of full comprehensive approaches to handle the analysis of the full PSG is also desirable to help the clinician with the time-consuming scoring task. These approaches may also contribute to reveal new data and patterns of relevance to improve the diagnosis. Some aspects to be improved within the current available approaches have to deal with the excessive sensitivity to the presence of artifacts and noise, the difficulty to handle variability present in the signals, excessive dependence on thresholds, lack of explanation of their results, or the bias due to patient dependency and human subjectivity. Recent developments in artificial intelligence and signal analysis may play an important role in the future development of SAHS diagnostic systems.

Further testing is also needed. To correctly assess the actual capabilities of the current approaches, validation has to be extended to increase the patient's sample, involve more heterogeneous cases, and make use of standard databases. Standardization of the validation process should be assessed as well in order to allow meaningful comparison of the results.

## Figures and Tables

**Table 1:** Summary of SAHS screening approaches.

Source	Method	References

Oxygen saturation	Counting on the number of desaturation events	Netzer et al. [16]
	Time spent below 90%	Netzer et al. [16]
	Expert systems	Daniels et al. [17]
	Delta-index	Lévy et al. [18], Olson et al. [19], and Magalang et al. [20]
	Spectral analysis	Zamarrón et al. [21, 22], Hua and Yu [23], and Morillo et al. [24]
	Nonlinear methods (CTM, LZ, and ApEn)	Álvarez et al. [25, 26], Hornero et al. [27], and del Campo et al. [28]
	Machine learning	Victor-Marcos et al. [29–33], Álvarez et al. [34, 35], and El-Solh et al. [36]

Airflow	Spectral analysis	Gutierrez-Tobal et al. [37]
	Nonlinear methods (CTM, LZ, and ApEn)	Gutiérrez-Tobal et al. [38]
	Hilbert-Huang decomposition	Caseiro et al. [39], Salisbury and Sun [40]

**Table 2:** Validation of SAHS screening approaches (see text for details). *n* = number of participants/recordings; AHI = Apnea-Hypopnea Index; TR = training set; TS = testing set; H = hypopnea; PPV = positive predictive value; NPV = negative predictive value; AUC = areas under ROC curve; ACC = accuracy; ICC = intraclass correlation coefficient; *r* = Pearson's linear correlation coefficient; S = single validation set; CV = cross-validation; CSR-CSA = Cheyne-Stokes respiration.

Study source	Database	*n*/ratio male	Age (mean ± std.)	AHI (mean ± std.)	Reference test	Event definition	AHI cut-off	Sensitivity (%)	Specificity (%)	Others	Validation
Daniels et al. [17]	Birmingham Heartlands Hospital, UK	21/—	—	—	Retrospective clinical diagnosis	—	—	100	66	PPV = 0.95 NPV = 1.00	S

Lévy et al. [18]	CHU de Grenoble, France	301/—	56 ± 12	30 ± 24	Same-night Level 1 PSG	H: >50% reduction + [3% desaturation or arousal]	15	90	75	PPV = 0.87 NPV = 0.81	S

Olson et al. [19]	Newcastle Sleep Disorders Center, Australia	793/—	—	—	Same-night Level 1 PSG	—	5	82.7	54.2	—	S

Magalang et al. [20]	Associated Sleep Center and the Buffalo Veterans Affairs Medical Center, USA	224/— (TR) 101/— (TS1) 191/— (TS2)	48.9 ± 12.3 (TR) 51.8 ± 11.5 (TS1) 56.0 ± 12.8 (TS2)	18.2 ± 20.0 (TR) 20.2 ± 19.5 (TS1) 18.2 ± 21.2 (TS2)	Same-night Level 1 PSG	H: discernible reduction + [4% desaturation or arousal]	15	90 (TR) — (TS1) — (TS2)	70 (TR) — (TS1) — (TS2)	—	TR/TS1/TS2

Zamarrón et al. [21]	Hospital General de Galicia, Spain	233/0.80	56.8 ± 13.1 (OSA) 56.1 ± 13.0 (Non-OSA)	40.1 ± 23.0 (OSA) 2.2 ± 2.7 (Non-OSA)	Same-night Level 1 PSG	H: >50% reduction + 4% desaturation	10	78	89	PPV = 0.89 NPV = 0.78	S

Zamarrón et al. [22]	Hospital General de Galicia, Spain	300/0.78	58.4 ± 12.1 (OSA) 56.6 ± 12.8 (Non-OSA)	40.2 ± 22.4 (OSA) 2.3 ± 2.7 (Non-OSA)	Same-night Level 1 PSG	H: >50% reduction + 4% desaturation	10	94	82	PPV = 0.87 NPV = 0.92	S

Hua and Yu [23]	Chang Gung Memorial Hospital, China	273/0.77 (TR) 206/0.81 (TS)	45.8 ± 16.5 (TR) 46.2 ± 13.4 (TS)	31.6 ± 27.1 (TR) 44.1 ± 29.8 (TS)	Same-night Level 1 PSG	AASM1999 H: >50% reduction or <50% reduction + [3% desaturation or arousal]	5	—	—	AUC = 0.96	TR/TS

Morillo et al. [24]	University Hospital Puerta del Mar de Cadiz, Spain	37/0.78 (TR) 78/0.74 (TS)	57.9 ± 9.8 (TR) 58.3 ± 12.5 (TS)	32.6 ± 29.24 (TR) 28.8 ± 28.3 (TS)	Same-night Level 1 PSG	H: >50% reduction + 4% desaturation	20	86.4	82.8	AUC = 0.92 ICC = 0.82	TR/TS

Álvarez et al. [25, 26]	Hospital Clinico Universitario in Santiago de Compostela, Spain	111/0.85 (OSA) 76/0.70 (Non-OSA)	58.3 ± 12.9 (OSA) 57.6 ± 12.9 (Non-OSA)	40.1 ± 19.6 (OSA) 2.04 ± 2.36 (Non-OSA)	Same-night Level 1 PSG	H: reduction (no cessation) + 4% desaturation	10	90.1	82.9	AUC = 0.92 ACC = 0.87	S

Hornero et al. [27]	Hospital Clinico Universitario in Santiago de Compostela, Spain	111/0.85 (OSA) 76/0.70 (Non-OSA)	58.3 ± 12.9 (OSA) 57.6 ± 12.9 (Non-OSA)	40.1 ± 19.6 (OSA) 2.04 ± 2.36 (Non-OSA)	Same-night Level 1 PSG	H: >50% reduction + 4% desaturation	10	82.1	87	—	TR/TS (113)

del Campo et al. [28]	Hospital Clinico Universitario in Santiago de Compostela, Spain	111/0.85 (OSA) 76/0.70 (Non-OSA)	58.3 ± 12.9 (OSA) 57.6 ± 12.9 (Non-OSA)	40.1 ± 19.6 (OSA) 2.03 ± 2.19 (Non-OSA)	Same-night Level 1 PSG	H: >50% reduction + 3% desaturation	10	88.3	82.9	AUC = 0.92 PPV = 0.88 NPV = 0.83	S

Marcos et al. [29]	Hospital Clinico Universitario in Santiago de Compostela, Spain	111/0.85 (OSA) 76/0.70 (Non-OSA)	58.3 ± 12.9 (OSA) 57.6 ± 12.9 (Non-OSA)	40.1 ± 19.6 (OSA) 2.04 ± 2.36 (Non-OSA)	Same-night Level 1 PSG	H: >50% reduction + 4% desaturation	10	79.1	97.8	AUC = 0.92 ACC = 0.87	TR/TS (113)

Victor-Marcos et al. [30]	Hospital Universitario Pío del Río Hortega, Spain	166/0.84 (OSA) 48/0.67 (Non-OSA)	55 ± 13.5 (OSA) 47.7 ± 9.76 (Non-OSA)	37.1 ± 25.8 (OSA) 4.13 ± 2.39 (Non-OSA)	Same-night Level 1 PSG	H: >50% reduction + 4% desaturation	10	97.0	79.3	AUC = 0.95 ACC = 0.93	TR/TS (129)

Álvarez et al. [34]	Hospital Universitario Pío del Rio Hortega, Spain	100/0.84 (OSA) 48/0.67 (Non-OSA)	55.2 ± 14.6 (OSA) 48.3 ± 11.8 (Non-OSA)	40.9 ± 27.6 (OSA) 4.1 ± 2.4 (Non-OSA)	Same-night Level 1 PSG	H: >50% reduction + [3% desaturation or arousal]	10	92	85.4	AUC = 0.97 ACC = 0.90	CV (Leave-one-out)

Álvarez et al. [35]	Hospital Universitario Pío del Rio Hortega, Spain	160/0.84 (OSA) 80/0.65 (Non-OSA)	54.8 ± 13.8 (OSA) 47.2 ± 12.2 (Non-OSA)	36.6 ± 25.7 (OSA) 3.9 ± 2.4 (Non-OSA)	Same-night Level 1 PSG	AASM2007 H: >50% reduction + [3% desaturation or arousal]	10	90.6	81.3	ACC = 0.87	TR/TS (144)

El-Solh et al. [36]	Veterans Affairs Medical Center, USA	213/—	—	—	Same-night Level 1 PSG	H: discernible reduction + [3% desaturation or arousal]	5 (OSA) 10 (CSR-CSA)	97.7 (OSA) 100 (CSR-CSA)	100 (OSA) 99 (CSR-CSA)	ACC = 0.99 (OSA, CSR-CSA)	CV (5 K-fold)

Marcos et al. [31]	Hospital Clinico Universitario in Santiago de Compostela, Spain	111/0.85 (OSA) 76/0.70 (Non-OSA)	58.3 ± 12.9 (OSA) 57.6 ± 12.9 (Non-OSA)	40.1 ± 19.6 (OSA) 2.04 ± 2.36 (Non-OSA)	Same-night Level 1 PSG	H: >50% reduction + 4% desaturation	10	89.4 ± 1.6	81.4 ± 1.7	AUC = 0.91 ACC = 0.86	TR/TS1 (30)/TS2 (83)

Victor-Marcos et al. [32]	Hospital Clinico Universitario in Santiago de Compostela, Spain	111/0.85 (OSA) 76/0.70 (Non-OSA)	58.3 ± 12.9 (OSA) 57.6 ± 12.9 (Non-OSA)	40.1 ± 19.6 (OSA) 2.04 ± 2.36 (Non-OSA)	Same-night Level 1 PSG	H: >50% reduction + 4% desaturation	10	90.8 ± 4.2	79.1 ± 6.9	AUC = 0.91 ACC = 0.86	TR/TS1 (30)/TS2 (83)

Victor-Marcos et al. [33]	Hospital Universitario Pío del Rio Hortega, Spain	240/0.77	52.3 ± 13.8 (TR) 52.2 ± 13.7 (TS)	24.7 ± 25.2 (TR) 26.4 ± 26.7 (TS)	Same-night Level 1 PSG	—	15	94.9	90.9	ACC = 0.93 ICC = 0.91	TR/TS (144)

Gutierrez-Tobal et al. [37]	Hospital Universitario Pío del Rio Hortega, Spain	148/0.79	50.9 ± 11.7	23.5 ± 24.1	Same-night Level 1 PSG	H: >30% reduction + 4% desaturation	30	76.7	93.2	ACC = 0.88	TR/TS (89)

Gutiérrez-Tobal et al. [38]	Hospital Universitario Pío del Rio Hortega, Spain	148/0.79	50.9 ± 11.7	23.5 ± 24.1	Same-night Level 1 PSG	H: >30% reduction + 4% desaturation	10	88	70.8	AUC = 0.90 ACC = 0.82	CV (Leave-one-out)

Caseiro et al. [39]	Coimbra Sleep Disorders Clinic, Portugal	21/0.52 (OSA) 20/0.35 (Non-OSA)	59.9 ± 13.9 (OSA) 40.8 ± 12 (Non-OSA)	47.4 ± 32.8 (OSA) 2.17 ± 1.56 (Non-OSA)	Same-night Level 1 PSG	—	5	81	95	AUC = 0.88	S

Salisbury and Sun [40]	Vanguard Technologies	18/— (TR1) 16/— (TR2)	—	— (TR1) 33.2 ± 35.7 (TR2)	Retrospective clinical diagnosis (TR1) Different-night Level 1 PSG (TR2)	H: >50% reduction + 5% desaturation	5	100 (TR1) 85.7 (TR2)	100 (TR1) 100 (TR2)	*r* = 0.30	S

**Table 3:** Summary of apneic event detection approaches.

Category	Source	Method	References
Pulse oximetry	SaO_2_	Counting on the number of desaturation and/or resaturation events	Netzer et al. [16], Flemons et al. [42]
		Online processing + Bagging classifier	Burgos et al. [43]
	PPG	DAP detector based on PPG	Gil et al. [44]

Single-channel respiratory activity	Abdominal breathing signal	Expert system	Macey et al. [45]
	NAF	Amplitude based thresholding	Reisch et al. [46], Pépin et al. [47]
		ANN	Várady et al. [48]
		Mean magnitude of the second derivative	Han et al. [49]
		Flow-power dips, amplitude based detection, and ODI	Nakano et al. [50]
		Fuzzy Inference System	Shin et al. [51], Nazeran et al. [52], Morsy and Al-Ashmouny [53], and Al-Ashmouny et al. [54]
	Thoracic breathing signal	Amplitude based thresholding	Reisch et al. [46]
	Esophageal pressure	Amplitude based thresholding	Reisch et al. [46]
	FOT	Amplitude based thresholding	Reisch et al. [46]

Multichannel respiratory activity	SaO_2_ + RIP	Amplitude based analysis	Taha et al. [55]
	Airflow + SaO_2_	TDNN	Tian and Liu [56]
	NAF + PPG	Pulse wave amplitude analysis	Sommermeyer et al. [57]
	Nasal pressure + FOT	Amplitude based feature extraction + thresholding	Steltner et al. [58]
	Thoracic and abdominal respiratory signals	Piecewise liner approximation + time-domain phase difference detection	Várady et al. [59]
	NAF + thoracic and abdominal breathing	Breath-to-breath amplitude analysis	Houdt et al. [60]
	Thoracic and abdominal respiratory effort + ECG + SaO_2_	SVM	Al-Angari and Sahakian [61]
	EEG + ECG + nasal pressure + oronasal temperature + right EOG + EMG	Wavelets + LAMSTAR ANN	Waxman et al. [62, 63]
	Airflow + SaO_2_ + thoracic and abdominal breathing	Fuzzy Inference System	Álvarez-Estévez and Moret-Bonillo [64], Maali and Al-Jumaily [65], and Otero et al. [66, 67]

**Table 4:** Validation of SAHS detection approaches (see text for details). *n* = number of participants/recordings; AHI = Apnea-Hypopnea Index; TR = training set; TS = testing set; A = apnea; H = hypopnea; N = normal breathing; PPV = positive predictive value; NPV = negative predictive value; AUC = areas under ROC curve; ACC = accuracy; ICC = intraclass correlation coefficient; *κ*_*w*_ = weighted Kappa coefficient; *r* = Pearson's linear correlation coefficient; DU = detection unit; C = continuous; S = single validation set; CV = cross-validation.

Study source	Database	*n*/ratio male	Age (mean ± std.)	AHI (mean ± std.)	Reference test	Event definition	Number of events	DU	Sensitivity (%)	Specificity (%)	Others	Validation
Burgos et al. [43]	Apnea-ECG [68] (Physionet [69])	8/0.87	43.2 ± 8.34	31.9 ± 35.9	Same-night Level 1 PSG	H: >50% reduction + 4% desaturation + compensatory hyperventilation	2465 (TR) 1498 (TS)	60 s	92.3	93.5	AUC = 0.98	TR/TS

Gil et al. [44]	Apnea-ECG [68] (Physionet [69]) (TR1) Miguel Servet Children's Hospital, Spain (TR2)	8/0.87 (TR1) 26/— (TR2)	43.2 ± 8.34 (TR1) Children (TR2)	32 ± 35.9 (TR1) — (TR2)	Same-night Level 1 PSG	H: discernible reduction + 3% desaturation	1609 (TR1) 207 (TR2)	C	95.3 (TR1) 74 (TR2)	—	PPV = 0.94 (TR1)	S

Macey et al. [45]	Christchurch Hospital, New Zealand	10/— (TR) 85/— (TS1) 57/— (TS2)	Infants	—	Same-night Level 1 PSG (TR) Same-night Level 2 PSG (TS1, TS2)	Only apneas scored: flat region > 5 s	619 (TR) 28339 (TS1) 32222 (TS2)	C	94.1 (TS1) 96.1 (TS2)	60.9 (TS1) 59.2 (TS2)	—	TR/TS1/TS2

Reisch et al. [46]	Ambrock Hospital, Germany	51/0.90	51.2	9.1	Same-night Level 1 PSG	See [46]	500	C	25–94	87–100	—	TR/TS (250)

Várady et al. [48]	MIT-BIH [70] (Physionet [69])	16/—	—	—	Same-night Level 1 PSG	—	8000 epochs	16 s	98.4 (N) 78.7 (H) 97.0 (A)	94.0 (N) 91.0 (H) 88.7 (A)	—	TR/TS

Han et al. [49]	Center for Sleep and Chronobiology of Seoul National University Hospital, South Korea	24/1.00	50.2 ± 11.1	37.7 ± 20.5	Same-night Level 1 PSG	—	195.5 ± 94.3 (A)	1 s	92.4 ± 5.5	88.3 ± 5.8	ACC = 0.92	TR/TS (21 P)

Pépin et al. [47]	Grenoble University Hospital, France	29/0.69	47 ± 15 (TR) 47 ± 12 (TS)	21.6 ± 18.5 (TR) 20.4 ± 18.8 (TS)	Same-night Level 1 PSG	H: >50% reduction or 30–50% reduction + [3% desaturation or arousal]	—	C	—	—	—	TR/TS (19 P)

Nakano et al. [50]	Fukuoka National Hospital, Japan	100/0.79 (TR) 299/0.85 (TS1) 119/— (TS2)	51.1 ± 13.7 (TR) 48.6 ± 13.9 (TS1) — (TS2)	24.4 ± — (TR) 25.6 ± — (TS1) — (TS2)	Same-night Level 1 PSG	H: >50% reduction or <30–50% reduction + [3% desaturation or arousal]	2744 (TS1) 2420 (TS2)	C	77 (TS1) 89 (TS2)	91 (TS1) 86 (TS2)	—	TR/TS1/TS2

Shin et al. [51]	—	13/1.00	56.8 ± 1.9	—	Same-night Level 1 PSG	H: >50% reduction	—	C	—	—	*r* = 0.86	S

Nazeran et al. [52]	Texas Southwestern Medical Center, USA	9/0.78	48 ± 15	39 ± 29	Same-night Level 1 PSG	—	808 (A)	C	83	—	ACC = 0.83	S

Morsy and Al-Ashmouny [53]	—	10/—	—	—	—	—	—	C	99.9 ± 0.0	96.6 ± 0.04	—	TR/TS

Al-Ashmouny et al. [54]	—	30/—	—	—	—	—	3626	C	—	—	—	S

Taha et al. [55]	Wisconsin Sleep Cohort Study [71]	10/—	51.8 ± 7.17	18.2 ± 16.2	Same-night Level 1 PSG	H: discernible reduction + 2% desaturation	1804 (SDB)	C	93.1	—	PPV = 0.97	S

Tian and Liu [56]	—	30/—	—	—	Same-night Level 1 PSG	H: >50% reduction + 3% desaturation	302 (A) 728 (H)	1 s	90.7 (A) 80.8 (H)	86.4 (A) 81.4 (H)	—	TR/TS (15 P)

Sommermeyer et al. [57]	Sahlgrenska University Hospital, Sweden, and sleep centers in Ulm, Berlin, and Nuremberg, Germany	66/0.63	54 ± 14	19.3 ± 18.5	Same-night Level 3 PG	H: >50% reduction + 3% desaturation	4715 (A) 4089 (H)	C	—	—	*r* = 0.95	S

Steltner et al. [58]	Freiburg University Hospital, Germany	19/1.00	59.9 ± 10.6	16.5 ± 13.29	Same-night Level 1 PSG	H: >50% reduction or discernible reduction + [3% desaturation or arousal]	3957 (scorer 1) 2966 (scorer 2)	C	75.1 (scorer 1) 85.5 (scorer 2)	—	*κ*_*w*_ = 0.45 ± 0.15 (scorer 1) 0.40 ± 0.19 (scorer 2)	CV (Leave-one-out P)

Várady et al. [59]	—	6/—	—	—	PSG (unknown level or timing)	—	189	C, val = 5 min	—	—	ACC = 0.91	S

Houdt et al. [60]	Sleep Medicine Center, Kempenhaeghe, The Netherlands	11/0.91	49.1 ± 11.4	16.5 ± 13.3	Same-night Level 1 PSG	AASM2007 H: >50% reduction + [3% desaturation or arousal]	299 (A) 504 (H) (scorer 1) 293 (A) 540 (H) (scorer 2)	30 s	89.2 (scorer 1) 88.9 (scorer 2)	—	PPV = 0.54 (scorer 1) 0.59 (scorer 2)	TR/TS (6 P)

Al-Angari and Sahakian [61]	Sleep Heart Health Study [72]	50/— (OSA) 20/— (Non-OSA)	—	36.9 ± 24.3 (OSA) 1.1 ± 1.48 (Non-OSA)	Same-night Level 2 PSG	H: >30% reduction or discernible reduction + 2% desaturation (see [4])	22908	60 s	69.9	91.4	ACC = 0.82	S

Waxman et al. [62, 63]	Sleep Center University of Illinois, USA	74/0.72	48.1 ± 10.8	36.8 ± 30.5	Same-night Level 1 PSG	AASM1999 H: >50% reduction or <50% reduction + [3% desaturation or arousal]	6371 (A) 1167 (H)	30 s	88.6 ± 1.2 (A in NREM) 82.8 ± 3.5 (H in NREM)	85.3 ± 1.2 (A in NREM) 77.2 ± 5.2 (H in NREM)	—	TR/TS

Álvarez-Estévez and Moret-Bonillo [64]	Sleep Heart Health Study [72]	12/—	—	—	Same-night Level 2 PSG	H: >30% reduction or discernible reduction + 2% desaturation (see [4])	4866	C, val = 30 s	87 92 (A) 85 (H)	89 85 (A) 92 (H)	—	S

Maali and Al-Jumaily [65]	—	2/—	—	—	—	—	959	C	—	—	ACC = 0.95	S

Otero et al. [66]	Hospital Clinico Universitario in Santiago de Compostela, Spain	12/—	—	—	Same-night Level 1 PSG	—	5796	C	95.7	98.4	ACC = 0.89	S

Otero et al. [67]	Hospital Clinico Universitario in Santiago de Compostela, Spain	10/—	—	—	Same-night Level 1 PSG	—	1706 (A)	C	90 (A)	96.8 (A)	—	S

**Table 5:** Summary of apneic event classification methods. S = snoring; H = hypopnea; N = normal breathing; OH = obstructive hypopnea; CH = central hypopnea; CA = central apnea; OA = obstructive apnea; MA = mixed apnea; N/A = not available.

Source	Method	Classes	References
NAF, thoracic breathing signal, esophageal pressure, and FOT	Amplitude based thresholding	H1, H2, OA, CA, and MA	Reisch et al. [46]

Airflow + thoracic and abdominal effort signals	Fuzzy Inference System	N, H, CA, and OA	Al-Ashmouny et al. [54]
	Self-advising SVM	CA, OA, and MA	Maali et al. [77]

SaO_2_ + RIP	Amplitude based analysis	N, H, OA, CA, and MA	Taha et al. [55]

PPG + NAF	Pulse wave amplitude analysis	OA, CA	Sommermeyer et al. [57]

Nasal pressure + FOT signal	Amplitude based feature extraction + thresholding	N, H, OA, CA, and MA	Steltner et al. [58]

Thoracic and abdominal respiratory signals	Piecewise linear approximation + time-domain phase difference detection	N, OA, and CA	Várady et al. [59]
	Multiresolution DWT + ANN	CA, OA, and MA	Sezgin and Emin Tagluk [78]

NAF + thoracic and abdominal breathing	Breath-to-breath amplitude analysis	N, H, OA, CA, MA, and N/A	Houdt et al. [60]

NAF + Pes + Pgas signals	Hidden Markov models	N, S, CA, and OA	Al-Ani et al. [79]

Airflow + thoracic effort signals	Amplitude analysis + wavelets + Bayesian ANN	N, CA, OA, and MA	Fontenla-Romero et al. [80]

Abdominal effort signal	Multiresolution DWT + ANN	CA, OA, and MA	Tagluk et al. [81, 82]

Thoracic effort signal	Discrete wavelet decomposition + SVM RFE feature selection + combination of ANNs	CA, OA, and MA	Guijarro-Berdiñas et al. [83]

NAF, Pes	Amplitude based features + Adaboost classifier (Pes); inspiratory flow limitation detector + feature extraction + diagonal quadratic discriminant (NAF)	OH, CH	Morgenstern et al. [84]

**Table 6:** Validation of respiratory event classification approaches (see text for details). *n* = number of participants/recordings; AHI = Apnea-Hypopnea Index; TR = training set; TS = testing set; A = apnea; H = hypopnea; N = normal breathing; CA = central apnea; OA = obstructive apnea; MA = mixed apnea; PPV = positive predictive value; NPV = negative predictive value; AUC = areas under ROC curve; ACC = accuracy; ICC = intraclass correlation coefficient; *κ*_*w*_ = weighted Kappa coefficient; *r* = Pearson's linear correlation coefficient; S = single validation set; CV = cross-validation. ^1^Minimum and maximum values.

Study source	Database	*n*/ratio male	Age (mean ± std.)	AHI (mean ± std.)	Reference test	Event definition	Number of events	Sensitivity (%)	Specificity (%)	Others	Validation
Reisch et al. [46]	Ambrock Hospital, Germany	51/0.90	51.2	9.1	Same-night Level 1 PSG	See [46]	50 (H1) 50 (H2) 50 (OA) 50 (MA) 50 (CA)	56–85 (H1) 74–85 (H2) 25–94 (OA) 82–88 (MA) 60–88 (CA)	90–98 (H1) 90–98 (H2) 96–100 (OA) 87–100 (MA) 96–100 (CA)	—	TR/TS

Al-Ashmouny et al. [54]	Cairo University, Egypt	—/—	—	—	—	—	3626	—	—	—	S

Taha et al. [55]	Wisconsin sleep cohort study [71]	10/—	51.8 ± 7.17	18.2 ± 16.2	Same-night Level 1 PSG	See [55]	142 (OA) 41 (CA) 7 (MA)	68.3 (OA) 75.6 (CA) 100 (MA)	96.0 (OA) 100 (CA) 77.5 (MA)	ACC = 0.71	S

Sommermeyer et al. [57]	Sahlgrenska University Hospital, Sweden, and sleep centers in Ulm, Berlin and Nuremberg, Germany	66/0.63	54 ± 14	19.3 ± 18.5	Same-night Level 3 PG	See [57]	3673 (OA) 1042 (CA)	—	—	*r* = 0.87 (OA) *r* = 0.95 (CA)	S

Steltner et al. [58]	Freiburg University Hospital, Germany	19/1.00	59.9 ± 10.6	16.5 ± 13.29	Same-night Level 1 PSG	See [58]	3957 (scorer 1) 2966 (scorer 2)	Scorer 1 = 60 (H) 63 (OA) 20 (MA) 37 (CA) Scorer 2 = 72 (H) 52 (OA) 0 (MA) 39 (CA)	—	*κ*_*w*_ = 0.45 ± 0.15 (scorer 1) 0.40 ± 0.19 (scorer 2)	CV (Leave-one-out P)

Várady et al. [59]	—	6/—	—	—	PSG (unknown level or timing)	—	189 (5 min each)	—	—	ACC = 0.91	S

Houdt et al. [60]	Sleep Medicine Center, Kempenhaeghe, The Netherlands	11/0.91	49.1 ± 11.4	16.5 ± 13.3	Same-night Level 1 PSG	AASM2007	—	—	—	ACC = 0.68	S

Al-Ani et al. [79]	Raymond Poincare Hospital, France	—/—	—	—	—	—	—	—	—	ACC = 0.90–1.00	—

Fontenla-Romero et al. [80]	Complejo Hospitalario Universitario de A Coruña, Spain	6/—	—	—	Same-night Level 1 PSG	—	40 (OA) 40 (MA) 40 (CA)	—	—	ACC = 0.81 (OA) 0.80 (MA) 0.90 (CA)	CV (100 × 10 K-fold)

Tagluk et al. [81]	Medicine Faculty of Dicle University, Turkey	21/0.71	21–67^1^	—	Same-night Level 1 PSG	—	120 (OA) 120 (CA) 120 (MA)	—	—	ACC = 0.73 (OA) 0.94 (CA) 0.66 (MA)	TR/TS (240)

Tagluk and Sezgin [82]	Medicine Faculty of Dicle University, Turkey	21/0.71	21–67^1^	—	Same-night Level 1 PSG	—	120 (OA) 120 (CA) 120 (MA)	—	—	ACC = 0.85 (OA) 0.94 (CA) 0.78 (MA)	TR/TS (240)

Sezgin and Emin Tagluk [78]	Medicine Faculty of Dicle University, Turkey	21/0.67	21–57^1^	—	Same-night Level 1 PSG	—	120 (OA) 120 (CA) 120 (MA)	—	—	ACC = 0.86 (OA) 0.95 (CA) 0.80 (MA)	TR/TS (240)

Guijarro-Berdiñas et al. [83]	Complejo Hospitalario Universitario de A Coruña, Spain	6/—	—	—	Same-night Level 1 PSG	—	40 (OA) 40 (MA) 40 (CA)	89.6 (OA) 92.2 (MA) 89.0 (CA)	97.1 (OA) 89.6 (MA) 98.6 (CA)	ACC = 0.95 (OA) 0.90 (MA) 0.95 (CA)	CV (100 × 10 K-fold)

Maali et al. [77]	Concord Hospital, Australia	—/—	—	—	—	—	—	—	—	ACC = 0.84	—

Morgenstern et al. [84]	Sleep Lab, Bethanien Hospital, Germany	28/—	52.6 ± 15.6	18.9 ± 18.5	Same-night Level 1 PSG	AASM1999	769 (H)	90	90	ACC = 0.90	CV (100 K-fold)

**Table 7:** Summary of comprehensive diagnostic systems.

System	Method	References
PSG-EXPERT	Processing of symbolic information + certainty factors	Fred et al. [85]
—	Data fusion of complex symbolic objects	Ugon et al. [86]
TASAS	Reasoning with symbolic objects using causal constraint temporal networks + handling of uncertainty	Fernández-Leal et al. [87, 88]
—	Automatic pattern discovery and rule generation on multichannel time series	Guimaraes et al. [89]
SAMOA	Signal processing + symbolic rule-based reasoning and intelligent diagnosis	Cabrero-Canosa et al. [90, 91]
MIASOFT	Signal processing + intelligent analysis of sleep macro- and microstructure and respiratory activity and diagnosis + reasoning mechanisms to support uncertainty in the decision process	Alvarez-Estevez et al. [92–96]

**Table 8:** Validation of comprehensive approaches (see text for details). *n* = number of participants/recordings; A = Apnea; H = Hypopnea; OH = Obstructive Hypopnea; CH = Central Hypopnea; MH = Mixed Hypopnea; CA = Central Apnea; OA = Obstructive Apnea; MA = mixed apnea; AUC = areas under ROC curve; ACC = accuracy; S = single validation set.

Study source	Database	*n*/ratio male	Age (mean ± std.)	AHI (mean ± std.)	Reference test	Event definition	Number of events	Sensitivity (%)	Specificity (%)	Others	Validation
Fernández-Leal and Moret-Bonillo [87]	Complejo Hospitalario Universitario de A Coruña, Spain	10/—	—	—	Same-night Level 1 PSG	System SAMOA [90, 91]	1243	91.3 (OA) 100 (CA) 83.7 (MA) 91.1 (OH) 100 (CH) 60.0 (MH)	93.6 (OA) 95.7 (CA) 99.8 (MA) 98.3 (OH) 98.7 (CH) 99.9 (MH)	AUC = 0.92 (OA) 0.98 (CA) 0.92 (MA) 0.95 (OH) 0.99 (CH) 0.80 (MH)	—

Guimaraes et al. [89]	—	3/—	—	—	Same-night PSG (unknown level)	—	—	76.2 (Events)	75.8 (Events)	—	S

Cabrero-Canosa et al. [90, 91]	Complejo Hospitalario Universitario de A Coruña, Spain	13/—	—	—	Same-night Level 1 PSG	—	1270	87.5 (Diagnosis/—)	100 (Diagnosis/—)	ACC = 0.94 (Events) 0.80 (O) 0.92 (C) 0.85 (M) 0.92 (Diagnosis/—)	S

Alvarez-Estevez [92], Moret-Bonillo et al. [93]	Sleep Heart Health Study [72]	26/0.69	68.5 ± 7.7	50.0 ± 30.5	Same-night Level 2 PSG	Sleep Heart Health Study, reading center manual of operations [4]	8705	81.3 (events) 89.2 (A) 89.0 (H) 83.8 (OA) 78.3 (CA) 100 (diagnosis/10)	91.6 (Events) 89.0 (A) 89.2 (H) 78.3 (OA) 83.8 (CA) 33 (diagnosis/10)	ACC = 0.89 (events) 0.89 (A) 0.89 (H) 0.82 (OA) 0.82 (CA) 0.92 (diagnosis/10)	S

**Table 9:** Summary table on validation studies of commercial approaches (see text for details); *n* = number of participants/recordings; AHI = Apnea-Hypopnea Index; L = lab; H = home; AUC = area under ROC curve; *r* = Pearson's correlation coefficient; *κ* = Kappa index; ICC = intraclass correlation coefficient. ^1^Manual editing of the automatic results was allowed.

System	Device type	Database	*n*/ratio male	Age (mean ± std.)	AHI (mean ± std.)	Reference test	Event definition	Lab/home	Cut-off AHI	Sensitivity (%)	Specificity (%)	Others	Study source
Morpheus I	I	Brigham and Women's Hospital, USA	31/0.71	44.3 ± 11.3	20.6 ± 23 (scorer 1) 22.5 ± 24.5 (scorer 2)	Same-night Level 1 PSG	H: >30% reduction + 4% desaturation	L	15	—	—	AUC = 0.98	Pittman et al. [105]

Somnolyzer 24x7	I	SIESTA database [106]	51/0.86	51 ± 10	45 ± 31	Same-night Level 1 PSG	—	L	—	—	—	*r* = 0.94	Woertz et al. [107]

α-Somnostar 4100	I	Hospital Mutua de Terrassa, Spain	28/0.75	50 ± 8.8	—	Same-night Level 1 PSG	H: significant reduction + [3% desaturation or arousal]	L	10	55	100	ICC = 0.81	Barreiro et al. [108]

Morpheus Hx	III	Brigham and Women's Hospital, USA	53/0.68	47.8 ± 11.3	15.4 ± 24	Same-night Level 1 PSG	AASM2007	L	15	100	92.7	*r* = 0.96	Amir et al. [109]

ARES	III	Murrieta Sleep Medical Center and Pomona Valley Hospital, USA	284/0.62	48	—	Same-night Level 1 PSG	—	L	10	97.4	85.6	κ = 0.85	Westbrook et al. [110]
			187/0.66	46	—	Different-night Level 1 PSG	—	H	30	79.4	100	κ = 0.77	Westbrook et al. [110]
		NYU Sleep Disorders Center, USA	92/—	—	—	Same-night Level 1 PSG	H: >50% reduction or discernible reduction + 4% desaturation	L	15	95^1^	94^1^	—	Ayappa et al. [111]
			86/—	—	—	Different-night Level 1 PSG	H: >50% reduction or discernible reduction + 4% desaturation	H	15	85^1^	91^1^	—	Ayappa et al. [111]
		Prince of Wales Hospital, The Chinese University of Hong Kong, China	132/0.75	47.8 ± 9.8 (men) 52.3 ± 12.2 (women)	41.6 ± 26.9 (men) 32.3 ± 22.9 (women)	Same-night Level 1 PSG	AASM1999 H: >50% reduction or discernible reduction + [3% desaturation or arousal]	L	5	89	100	AUC = 0.97	To et al. [112]

Somnocheck	III	University of Erlangen-Nuremberg, Germany	51/0.86	53.4 ± 12.9	23.9 ± 26.2	Same-night Level 1 PSG	H: >50% reduction + 4% desaturation	L	10	83	95	*r* = 0.83	Ficker et al. [113]

WristOx 3100	IV	Hospital Aleman, Argentina	154/0.77	50.8 ± 14.3	13.8	Same-night Level 1 PSG	H: discernible reduction + [3% desaturation or arousal]	L	5	89.2	94.2	AUC = 0.96	Nigro et al. [114]

WatchPAT 100	IV	Brigham and Women's Hospital, USA	30/0.63	47 ± 14.8	23 ± 23.9	Same-night Level 1 PSG	AASM1999 H: >50% reduction or discernible reduction + [3% desaturation or arousal]	L	20	90.9	84.2	*r* = 0.87	Ayas et al. [115]
		Technion Sleep Medicine Center, Israel	102/0.76	41.4 ± 15.2	—	Same-night Level 1 PSG	AASM1999 H: >50% reduction or discernible reduction + [3% desaturation or arousal]	L	20	—	—	*r* = 0.88 AUC = 0.87	Bar et al. [116]
		Brigham and Women's Hospital, USA	29/0.72	43.2 ± 10.8	31.6 ± 20.6	Same-night Level 1 PSG	AASM1999 H: >50% reduction or discernible reduction + [3% desaturation or arousal]	L	10	96	100	*r* = 0.89 ICC = 0.88 AUC = 0.96	Pittman et al. [117]
						Different-night Level 1 PSG	AASM1999 H: >50% reduction or discernible reduction + [3% desaturation or arousal]	H	15	96	100	*r* = 0.72 ICC = 0.72 AUC = 0.97	Pittman et al. [117]
		Skaraborg Sleep Study [118], Sweden	98/0.56	60 ± 6.7	25.5 ± 22.9	Same-night Level 2 PSG	H: >50% reduction or discernible reduction + [4% desaturation or arousal]	H	10	—	—	*r* = 0.90 AUC = 0.93	Zou et al. [119]

ApneaLink	IV	Prince of Wales Hospital, The Chinese University of Hong Kong, China	50/0.88	50 ± 11.8	—	Same-night Level 1 PSG	AASM1999 H: >50% reduction or discernible reduction + [3% desaturation or arousal]	L	5	100^1^	100^1^	AUC^2^ = 1.00	Ng et al. [120]
		—	59/0.49	57.3 ± 12	—	Same-night Level 1 PSG	AASM1999	L	15	90.9	94.6	AUC = 0.97	Erman et al. [121]
		Sleep Disorders Center, King Saud University, Saudi Arabia	95/0.61	46.3 ± 12.6	34.1 ± 32.4	Same-night Level 1 PSG	AASM2007 H: >50% reduction + [3% desaturation or arousal]	L	30	63	98	*r* = 0.88 AUC = 0.88	Bahammam et al. [122]
		Hospital Aleman, Argentina	90/0.77	49.6 ± 15.1	—	Same-night Level 1 PSG	H: >50% reduction	L	30	88.5	95.3	ICC = 0.88 *r* = 0.89 AUC = 0.92	Nigro et al. [123]
		The University of British Columbia Hospital, Canada	50/0.64	48.7 ± 12.6	30 ± 25.8	Same-night Level 1 PSG	AASM1999	L	10	95	90	AUC = 0.98 ICC = 0.96	Chen et al. [124]
		—	102/0.76	54.7 ± 13.3	—	Same-night Level 1 PSG	AASM1999	L	15	92	88.5	*r* = 0.95	Ragette et al. [125]
			131/0.72	59.1 ± 14.6	—	Different-night Level 1 PSG	AASM1999	H	5	91.8	76.5	*r* = 0.85	Ragette et al. [125]

Somnolter	III	—	90/0.67	55.4 ± 8.7	—	Same-night Level 1 PSG	H: >50% reduction or >30% reduction + [3% desaturation or arousal]	L	—	83.6	81.8	*r* = 0.95	Cheliout-Heraut et al. [126]

Embletta	III	Sleep Center, Royal Infirmary NHS Trust, Scotland, UK	39/0.82	46 ± 9	35.4 ± 5.5	Same-night Level 1 PSG	H: >50% reduction (on nasal pressure)	L	—	—	—	*κ* = 0.28	Dingli et al. [127]
			50/—	50 ± 11	29.2 ± 2.7	Different-night Level 1 PSG	H: >50% reduction (on thoracoabdominal movement)	H	—	—	—	*κ* = 0.1	Dingli et al. [127]

Embletta PDS	III	Prince of Wales Hospital, China	80/0.78	51.4 ± 11.9	21.6 ± 19.1	Same-night Level 1 PSG	H: >30% reduction + [4% desaturation or arousal]	L	15	88	95	*r* = 0.98	Ng et al. [128]

Lifeshirt	III	—	48/0.72	44	19.3 ± 24.6	Same-night Level 1 PSG	H: >30% reduction + [3% desaturation or arousal]	L	25	100^1^	97^1^	AUC^2^ = 0.99	Goodrich and Orr [129]
		Dallas Veterans Affairs Medical Center, USA	10/0.8	48.8 ± 14.2	28.2 ± 21.1	Same-night Level 1 PSG	H: >25% reduction + 3% desaturation	L	5	100	50	*r* = 0.82	Carter et al. [130]
						Different-night Level 1 PSG	H: >25% reduction + 3% desaturation	H	5	100	100	*r* = 0.96	Carter et al. [130]

BreasSC20	III	Hospital Virgen de la Arrixaca, Spain	48/—	—	28.2	Same-night Level 3 PG	H: >30% reduction + 4% desaturation	H	5	—	—	*κ* = 0.72	Ruiz-López et al. [131]
		Hospital Txagorritxu de Victoria-Gasteiz, Spain	60/0.77	51.6 ± 13.2	31 ± 27.6	Same-night Level 1 PSG	H: >50% reduction + [3% desaturation or arousal]	L	20	93.5	96.6	*κ* = 0.90 AUC = 0.98	Núñez et al. [132]

RUSleeping RTS	IV	Greater Pittsburgh Clinical Sleep Laboratory, USA	25/0.36	42.4 ± 12.9	20.6 ± 26.8	Same-night Level 1 PSG	AASM1999	L	5	83	100	*r* = 0.73 AUC = 0.94	Grover and Pittman [133]

Stardust II	III	—	62/0.67	45.6 ± 18.2	25.3 ± 21.4	Same-night Level 3 PG	AASM1999 H: >50% reduction or discernible reduction + [3% desaturation or arousal]	H	50	100	92.5	*κ* = 0.33 *r* = 0.95	Yin et al. [134]
